# Logistic growth of a surface contamination network and its role in disease spread

**DOI:** 10.1038/s41598-017-13840-z

**Published:** 2017-11-01

**Authors:** Hao Lei, Yuguo Li, Shenglan Xiao, Xinyan Yang, ChaoHsin Lin, Sharon L. Norris, Daniel Wei, Zhongmin Hu, Shengcheng Ji

**Affiliations:** 1 Department of Mechanical Engineering, The University of Hong Kong, Hong Kong, SAR China; 2 Environmental Control Systems, Boeing Commercial Airplanes, Everett, WA USA; 3 Beijing Aeronautical Science & Technology Research Institute of COMAC, Beijing, China

## Abstract

Surfaces and objects surround us, and touching them is integral to everyday life. Pathogen contaminated surfaces (fomites) are known to transmit diseases. However, little is known about the ways and speed at which surfaces become contaminated. We found that under certain conditions, the number of contaminated surfaces grows logistically, corresponding to possible rapid transmission of infection. In such a surface network, pathogen can be transmitted great distances quickly—as far as people move. We found that the surface contamination network in aircraft cabins exhibits a community structure, with small communities connected by the aisle seatback surfaces and toilets, which are *high-touch* surfaces. In less than two to three hours, most high-touch surfaces in the cabin are contaminated, and within five to six hours nearly all touchable surfaces are contaminated. During short haul flight, aisle passengers have higher fomite exposure. This closely matches the spatial infection pattern of one reported inflight norovirus outbreaks. Our model is generally applicable to other crowded settings. The commonly repeated advice to “wash hands frequently” may be replaced in future by more strategic advice such as “clean surfaces right now”, or advice based on who should wash their hands, and when.

## Introduction

In the built environment, contaminated surfaces and objects, also called fomites, play a key role in the transmission of respiratory and enteric viral infections, including the norovirus. The mechanism of the fomite route is thought to involve two steps. First, a person touches a contaminated surface or object with his or her hand, which transfers the virus to their hand. Second, that person touches their contaminated hand to susceptible sites on his or her body, which inoculates the site with the virus, creating the opportunity for infection. We know that contaminated surfaces and objects can transmit disease, and that discarding contaminated objects and improving surface cleaning and hand hygiene can decrease infection^1–3^. Perhaps 20% to 40% of nosocomial infections arise via the contaminated hands of healthcare workers^4,5^, and via *high-touch* environmental surfaces^6^. Gwaltney and Hendley^7^ showed that touching surfaces leads to rhinovirus infections and there have been reviews of fomite transmission of the norovirus^8^. Many outbreak reports also reveal the role of surface contamination^9^. However, unlike our understanding of the airborne and close contact routes, our understanding of the fomite route remains at an empirical level^10,11^.

An environmental surface or object can be initially contaminated by the deposition of virus particles from the air^12^ (greatest on upward-facing surfaces), direct emission through coughing, aerosolisation of enteric viruses due to vomiting and diarrhoea incidents, toilet flushing (highest on surfaces in close proximity to sources^13,14^), and hand touching (greatest on high-touch surfaces). Many studies exist on pathogen transfer between hands and surfaces; and on pathogen survival on surfaces and skin^15–17^. A hand contaminated with certain viruses can contaminate up to seven other surfaces^18^.

**Figure 1:**
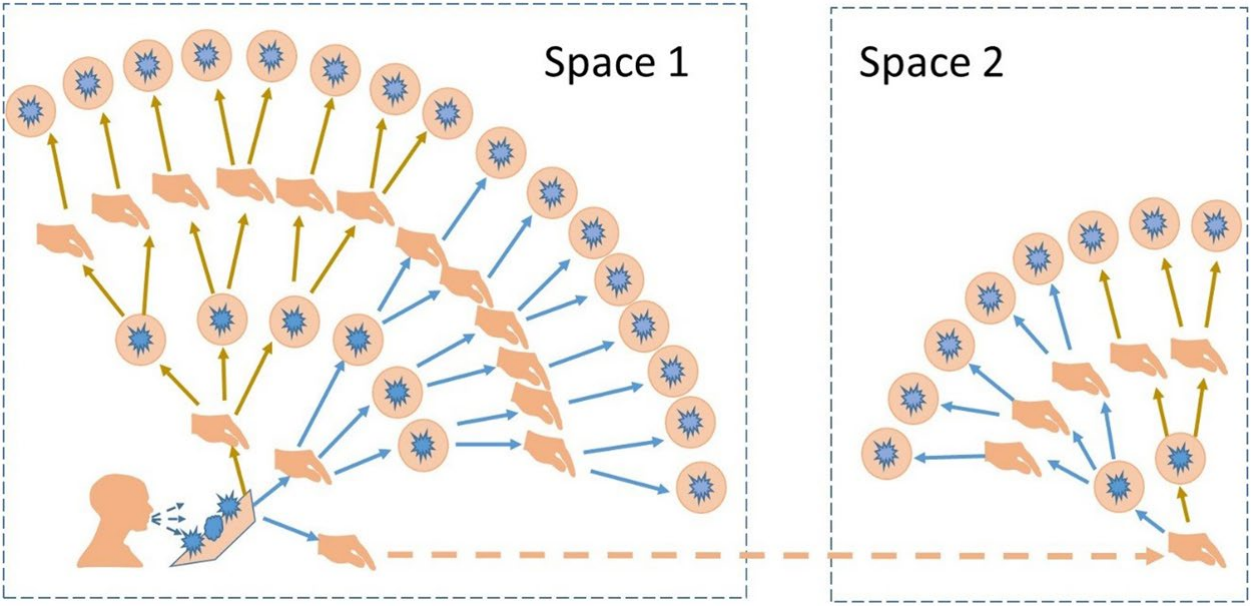
Illustration of how surfaces are connected to the root surface by hand touching. A contaminated hand may initiate a new network when an individual moves to a new environment.

Although the initial contamination process appears straightforward, there is limited information about how surface contamination is propagated by human touch. When we touch an object, we transfer viruses to a surface or accumulate more viruses on our hands. In a crowded environment, a surface (the ‘root surface’) contaminated by a source can be touched by a number of people, and each of these individuals subsequently touch other surfaces as they move around. Each of these now contaminated surfaces can be touched again by other people, and so the touching sequence progresses. Ultimately, all touched surfaces can be traced back to the root surface. We refer to this as the *surface touch network* (Fig. 1), which allows transfer of viruses and bacteria (or particles) between surfaces by hands, and results in a corresponding *surface contamination network*. We found that the growth of the number of contaminated surfaces is logistic. This is fundamental to the fomite transmission of diseases. The major physical processes involved in the fomite route include contamination of the root surface; virus survival on and transfer between the hand and surface during each subsequent touch; hand to mucous membrane transfer; and finally the dose-response, causing infection. In the literature, each of these processes has been studied and quantified to some extent for certain respiratory and enteric viruses, enabling us to focus on construction and analysis of the surface contamination network.

Existing social contact pattern studies in disease transmission are limited to close contact between people^19^. Mathematical studies for the fomite route exist^20^ and infection risk due to fomite transmssion has been calculated^20–24^. However, in all of these studies, the treatment of surface touching is simplified. These models cannot be used to study the spatial infection pattern of fomite transmission.

In this study, we networked environmental surfaces by connecting any two surfaces if they were sequentially touched by the same individual’s hands. The model was used to study infection transmission via the fomite route and to re-examine two inflight norovirus outbreaks. We have focused on the norovirus, for which the fomite route is known. As a non-enveloped virus, the norovirus is fairly stable on environmental surfaces^11^.

### Environmental setting

People’s surface touching behaviour varies in different environments. Aircraft cabins were considered in this study. In most other settings, such as classrooms, offices and hospitals, detailed spatial patterns for the secondary cases are generally not available when major outbreaks occur. The relatively fixed seating arrangement in aircraft cabins permits a spatial pattern of the secondary cases to be identified in some outbreaks. The temporal and spatial variation of climate and environment in aircraft cabins is also not as great as in other spaces. A number of inflight outbreaks have been well investigated, with detailed data available in the literature.

We simulated two reported inflight outbreaks of noroviruses^25,26^. The first was a norovirus genogroup II (GII) outbreak that occurred on 8 October 2008 during a three hour Boeing 737 flight from Boston, USA to Los Angeles, USA. Six of 35 tour group members reported experiencing vomiting or diarrhoea. Five GII passengers vomited into sickness bags while in their seats. After the flight, 7 of the 82 (attack rate, 8.5%) interviewed non-tour group passengers met the secondary case definition of norovirus illness^25^. The second was a norovirus genogroup I (GI) outbreak on a 12.5 hour Boeing 747–400 flight from Los Angeles, USA to Auckland, New Zealand on 19 January 2007. Two passengers had norovirus GI illness. After the flight, 52 of the 222 (attack rate, 23.4%) passengers interviewed met the secondary case definition of the norovirus illness^26^. The detailed environmental setting and spatial distribution of passengers in the two outbreaks are shown in Supplementary Information Figure S1.

### Characteristics of the surface contamination network

**Figure 2:**
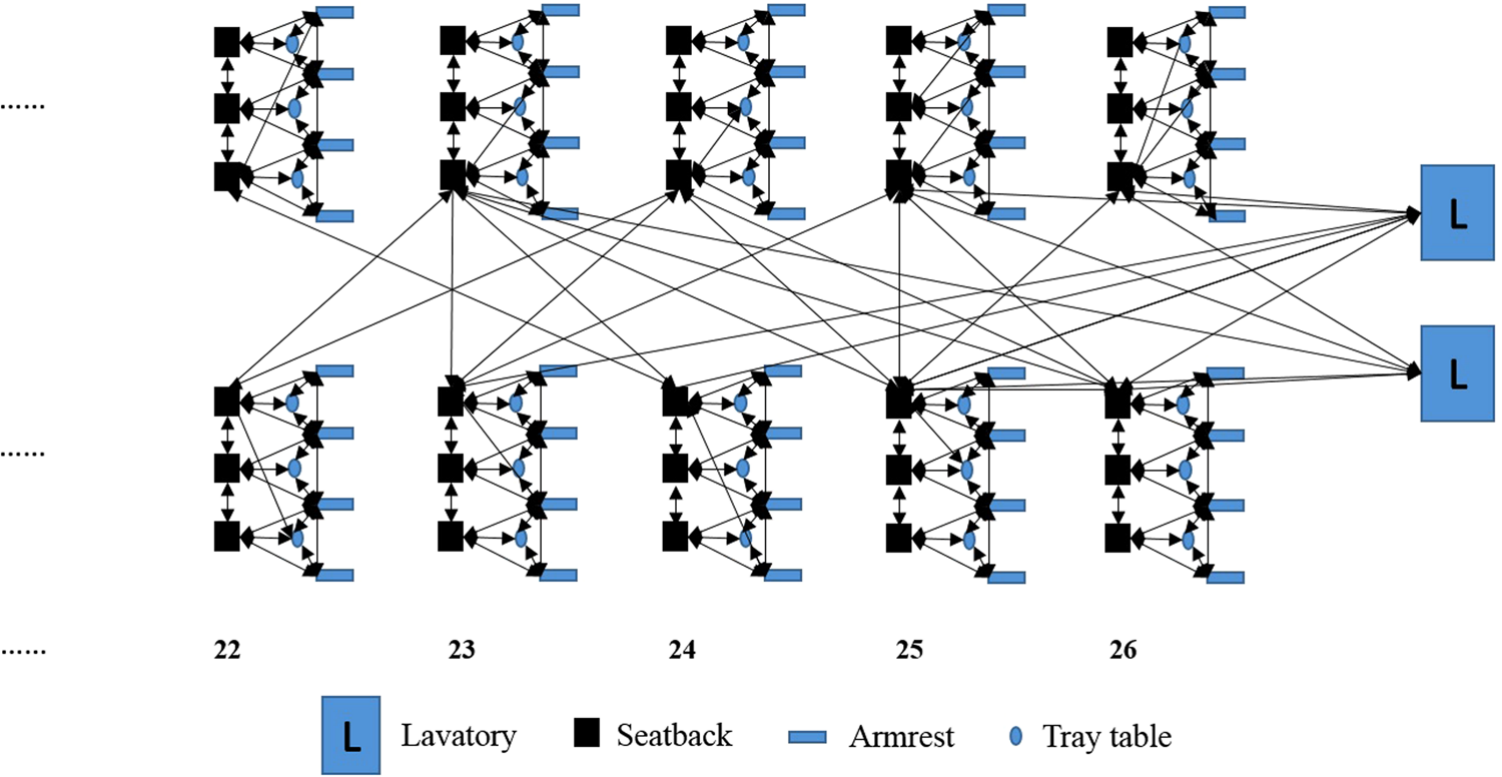
Part of the surface contamination network in one simulation of the GII 737 outbreak.

In a surface contamination network, any two sequentially touched environmental surfaces by the same individual are directly connected. As an example, we sketched a part of the surface contamination network (the toilets and the next five seat rows) in the Boeing 737 cabin in a simulation of the GII 737 outbreak; see Fig. 2. The network seems to exhibit a community structure. In this community network, the surfaces in half of a row on one side of the aisle form a sub-community, including three seatback surfaces, four armrest surfaces and three tray table surfaces. The surfaces in each of these sub-communities are densely connected locally. All sub-communities are connected by the aisle seatback surfaces, which also connect the toilet surfaces. Such a global connection exists because passengers may touch the aisle seatback surfaces when walking to the toilets, especially when the plane encounters turbulence.

As connections between the different sub-communities, the aisle seatbacks, which are high-touch surfaces, play an important role in the network. We analysed the properties of the surface contamination network on two levels: the aisle seatback surface contamination network, which only considers the aisle seatback surfaces and the surface contamination network, which includes all the surfaces. For this study, only Economy class in the Boeing 737 cabin with 21 seat rows was considered, and only Zones C and D with 24 seat rows were considered in the Boeing 747–400 cabin (Supplementary Information Figure S1). In the latter, the connection between the two zones is the toilets between them, because the passengers in Zone C and Zone D use the same toilets. Major properties of the surface contamination network are summarised in Table 1. To account for randomness, all the simulation cases were run 100 times, and the average value and the 95% confidence interval are shown in the Table. In the 737 Economy class, we considered 422 surfaces, including 42 aisle seatback surfaces and 84 non-aisle (21 × 6–42) seatback surfaces (the seatback surface on seats 21 A, 21B, 21E and 21 F would not be touched), 126 (21 × 6) tray table surfaces, 168 (21 × 8) armrest surfaces and 2 toilets. Zones C and D of the 747 aircraft have a total of 595 surfaces, including 71 aisle seatback surfaces.

**Table 1:** Properties of the aisle seatback surface contamination network and surface contamination network in the aircraft cabins in the GII 737 and GI 747 outbreaks.

		Diameter [95% CI]	Average geodesic distance [95% CI]	Average clustering coefficient [95% CI]
Aircraft cabin in the GII 737 outbreak	Aisle seatback surface contamination network	4.6 [4.23, 4.97]	2.04 [1.96, 2.11]	0.27 [0.25, 0.29]
	Entire surface contamination network	13.50 [13.00, 14.00]	5.38 [5.28, 5.48]	0.57 [0.56, 0.57]
Aircraft cabin in the GI 747 outbreak	Aisle seatback surface contamination network	4.90 [4.49, 5.31]	2.0 [2.00, 2.00]	0.52 [0.51, 0.52]
	Entire surface contamination network	7.90 [7.67, 8.12]	3.88 [3.85, 3.90]	0.66 [0.65, 0.66]

According to Rheinbaben *et al*.^18^, when 10^7^ plaque forming units of viruses are applied to door handles or hands, transmission of viruses from one person to another can be successful up to the sixth contact person. Barker *et al*.^1^ also showed that contaminated fingers can transfer noroviruses to as many as seven sequentially touched clean surfaces. Both studies reveal that viruses from one contaminated surface can be transferred to as many as six sequentially touched surfaces by one hand, when the hand is also considered to be a surface. In both the 737 and 747 cabins, the aisle seatback surface contamination network has a diameter of less than 5 edges, and this diameter is the longest of all the calculated shortest paths in the network. The small network diameter suggests that all the aisle seatback surfaces have the potential to become contaminated. The diameter of the entire surface contamination network is larger. The diameter of a small sub-community depends on whether the passengers go to the toilet; if all three passengers in a row sit in their seats during the flight, and only touch the front seatback, tray table and armrest surfaces, the diameter of the sub-community is only 4, such as the sub-community to the right of the 26th row (facing the toilet) in Fig. 2. However, if a passenger in a window seat goes to the toilet several times, he or she may touch the aisle seat just after touching the armrest surface close to the window and the diameter of the sub-community will be smaller. The flight duration in the GII outbreak was only three hours, whereas that of the GI outbreak was 12.5 hours. In both flights, we assumed that toilet use frequency was 1/6 per hour for susceptible individuals: half the passengers use the toilet during the flight in the Boeing 737, and each passenger uses the toilets twice during the flight in the Boeing 747. This explains why the average diameter of the surface contamination network is 13.5 in the 737 aircraft cabin, whereas in the 747 aircraft it is only 7.9.

### Logistic growth of the number of contaminated surfaces

The following assumptions were made in counting the number of contaminated surfaces: (1) once contaminated, environmental surfaces or hands remain contaminated unless these surfaces are sanitised, thus losing a proportion of their contamination dependent on the efficiency of sanitation; (2) some proportion of human norovirus particles on hands (environmental surfaces) is transferred to environmental surfaces (hands); (3) virus particles on hands lose infectivity as a function of time.

**Figure 3:**
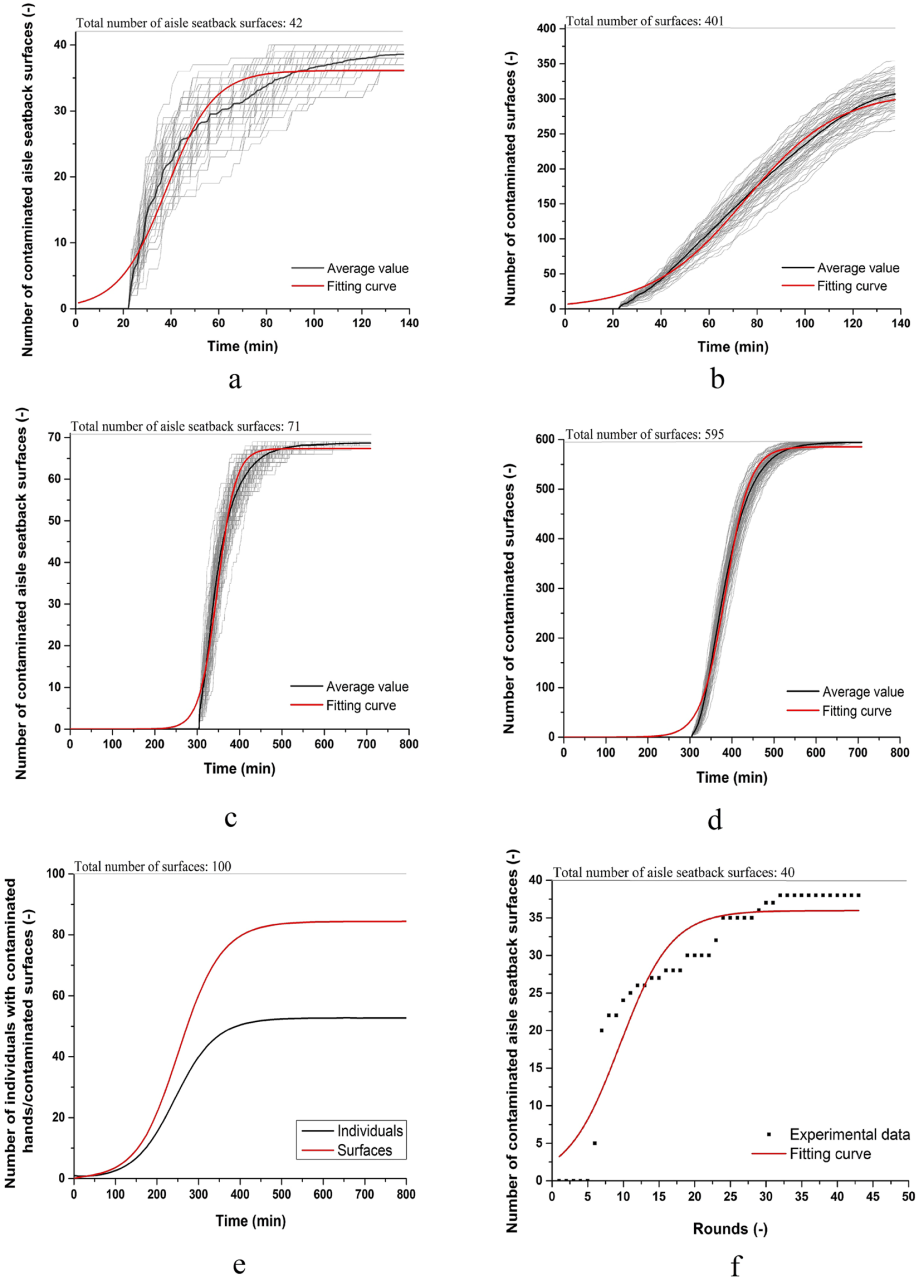
Growth of the number of contaminated surfaces (**a**). GII 737 outbreak, aisle seatback surfaces, (**b**) GII 737 outbreak, all surfaces; (**c**) GI 747 outbreak, aisle seatback surfaces, (**d**) GI 747 outbreak, all surfaces; (**e**) predicted result for both surfaces and individuals using a theoretical model shown in Supplementary Section 1.2; and (**f**) measured results (Supplementary Section 1.3). In (**a**–**d**), each graph shows 100 simulations (grey) together with the average of these 100 simulations (black), and the fitting curve using the logistic function (red).

Vomiting and diarrhoea are typical symptoms of norovirus gastroenteritis, so the time for the first passenger to vomit and then go to the toilet is also the time when the norovirus is emitted into the aircraft cabin. In the GII outbreak, the time at which the five index passengers vomited was not available. So in simulating the average infection risk, the time of vomiting was randomly chosen from the cruise phase. In counting the number of contaminated surfaces, which were simulated 100 times, we assumed random times for toilet use for the six index passengers in the first simulation, and for the remaining 99 simulations, we assumed that the time of toilet use remained the same as in the first simulation. The growth of the number of contaminated aisle seatback surfaces and of all surfaces in the Boeing 737 in 100 simulations is shown in Fig. 3a and b respectively. The effect of different critical values of surface contamination is shown in Supplementary Information Figure S8. As shown in Fig. 3a and b, a logistic growth model fits the growth of the number of contaminated aisle seatback surfaces and all surfaces well. Logistic growth suggests that surfaces can be contaminated quickly in crowded environments, in which case virus particles are likely to spread rapidly if not controlled early enough.

In the GI outbreak in the twin-aisle cabin, there were 71 aisle seatbacks surfaces in Zones C and D (Supplementary Information Figure S1) and 4 toilets between the two zones. The time when the index passenger vomited was known, and was used as the initial time for the growth of contaminated surfaces. The growth of the number of contaminated aisle seatback surfaces and all 595 surfaces in 100 simulations for this flight is shown in Fig. 3c and d. The logistic growth trend existed both for the aisle seatback surfaces and for all surfaces.

The logistic growth of contaminated surfaces is fundamental to fomite transmission. We performed a theoretical analysis and a bench-top experimental study to demonstrate its existence (Fig. 3e,f, and Supplementary Information Sections 1.3 and 1.4). The growth of the number of contaminated surfaces can be curtailed by surface cleaning and hand washing (Supplementary Information Figure S2).

### The aisle passengers had the highest fomite exposure risk

As the leading cause of viral gastroenteritis^25^, human noroviruses can spread from infectious faeces and vomitus through airborne droplets, fomites, and person-to-person contact^27^. Microbiological data shows that projectile vomiting associated with norovirus infections may distribute up to 3 × 10^7^ virus particles as an aerosol with a total volume of about 30 ml^13^. The airborne droplets can also be inhaled and deposited in the upper respiratory tract, and subsequently swallowed with the respiratory mucus^28^. In the GI 747 outbreak, the passengers vomited in the aisles, so the airborne route was also considered. Here we assumed that a person vomiting in the aisle produced 3 × 10^7^ droplets with a size distribution the same as that from a cough as described by Atkinson and Wein^22^; data on the size distribution of vomited droplets is not available.

The mean norovirus GII cDNA concentration is 3 × 10^8^ genomes/g in faeces, and the mean norovirus GI cDNA concentration is 8.4 × 10^5^ cDNA genomes/g^29,30^. The mass of vomitus on the hand of one person after vomiting was found to be 10^−3^ g in one study^31^, and the concentration of faeces or vomitus on a toilet surface is 1 g/cm^2^ after vomiting or diarrhoea^32^. In the GII 737 outbreak, passengers vomited into sickness bags, and one restroom on the airplane was eventually closed after it became soiled by sick passengers, so we can reasonably assume that there were no droplets emitted from the bags. As only one type was detected in the samples from the case passengers in each outbreak^25,26^, we assumed that all passengers on each flight were infected by the same type of norovirus. There was no reported body contact between ill passengers and susceptible individuals, so only the fomite route was considered in the simulations.

**Figure 4:**
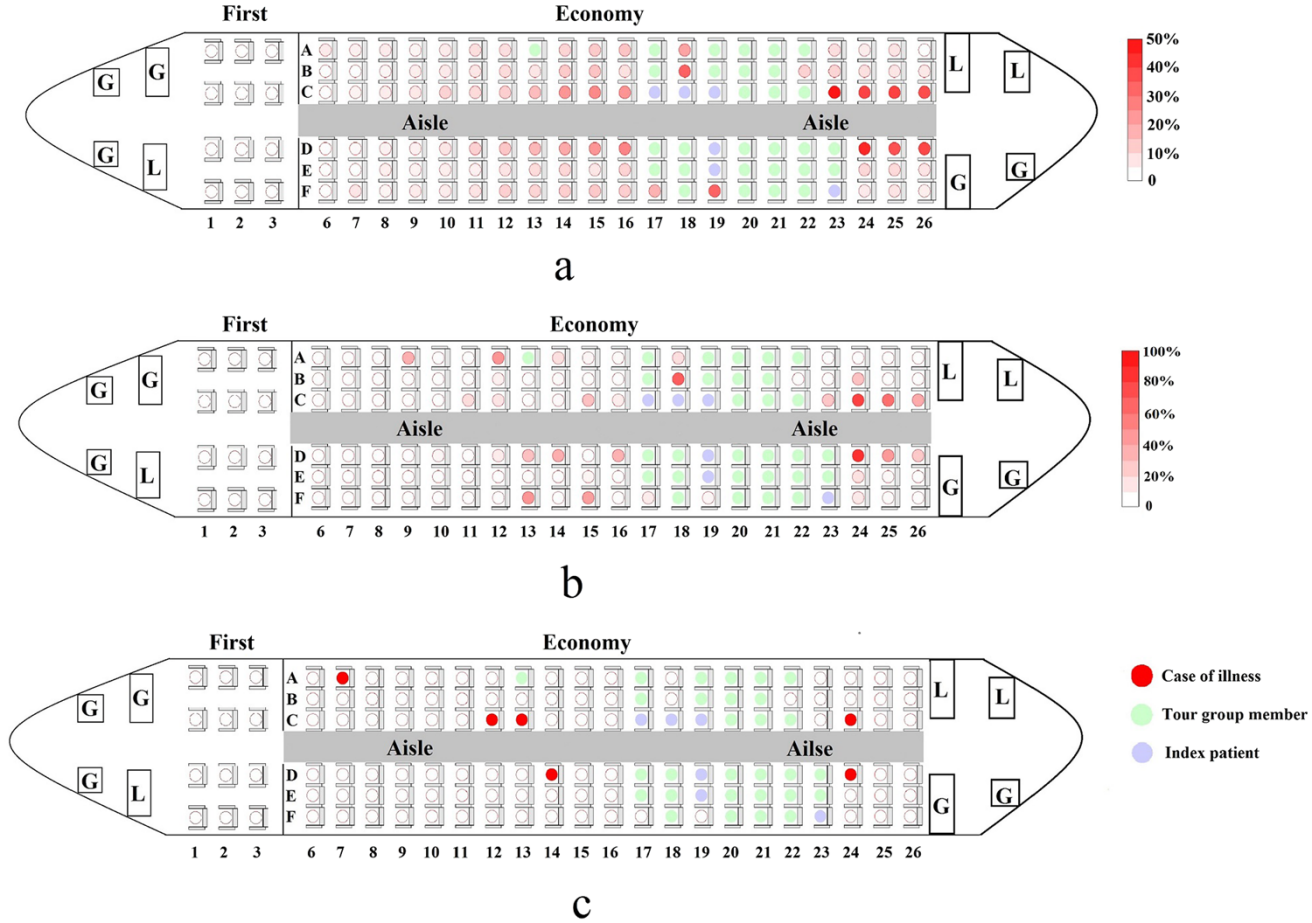
(**a**) Average simulated infection risk of 100 simulations; (**b**) simulated infection risk in one chosen simulation and (**c**) reported spatial distribution of cases in the norovirus GII 737 outbreak.

To take account of the randomness of the order in which passengers go to the toilet and touch aisle seatback surfaces, the infection risk per passenger varies even in the same condition in different simulations. We performed 100 simulations, and obtained the average infection risk of non-tour group members in Economy class by averaging the results from the simulations, as shown in Fig. 4a. Although it is not shown here, the predicted virus concentration on the aisle seatback surfaces is also higher than at other sites, which leads to a higher infection risk for aisle seat passengers than for non-aisle seat passengers. In addition, the predicted average infection risk of aisle seat passengers showed an inverse relationship with distance to the toilets.

What occurred in the reported outbreak (Fig. 4c) may not reflect the averaged infection risk as presented in Fig. 4a, but be closer to one of the simulations. It is conceivable that the attack rate did not exhibit a regular pattern as shown in Fig. 4a. After examining all 100 simulations, we considered one particular simulation result (shown in Fig. 4b). The infection risk in this simulation is very different from the averaged risks in Fig. 4a, but the spatial distribution is close to that reported; it is possible that some non-aisle seat passengers seated far away from the ill passengers have a high infection risk, such as the passengers seated in 13 F and 16 A in Fig. 4b. The occurrence of a transmission event can be random (e.g., these passengers may have used the toilet just after the ill passengers). This observation may explain why passenger 7 A was infected, as shown in Fig. 4c.

Table 2 compares the infection risk for the aisle and non-aisle seats for both the reported and predicted data for the two outbreaks. Our prediction fits the overall infection risk and the spatial distribution of individuals’ infection risks well. For the simulation results, both the average value and one particular case are presented. The flight duration in the GI 747 outbreak (12.5 hours) was much longer than that in the GII 737 outbreak (3 hours), which means that more surface touching behaviour will have occurred during the flight in the GI 747 outbreak. Therefore, more viruses would be transferred from high concentration surfaces (aisle seatback surfaces) to low concentration surfaces (non-aisle seatback surfaces, tray table and armrest surfaces). Note we assumed that viruses are transferred from high concentration to low concentration surfaces. The virus concentrations on different surfaces will then become more uniform. This is the main reason the relative risk for aisle seats in the GI 747 outbreak compared to non-aisle seats, defined as the ratio of the average infection risk of aisle seats to non-aisle seats (37.2 ÷ 31 = 1.2), is much lower than that in the GII 737 outbreak (20.8 ÷ 2 = 10.4). Another important reason is that in the GI 747 outbreak, the ill passengers vomited in the aisle in Zone C, which could have produced a large number of airborne droplets, leading to airborne transmission. Fewer airborne droplets were expected and predicted in Zone D, due to the curtain between Zones C and D. This leads to a higher relative risk for aisle seats compared to non-aisle seats in Zone D (1.8) than in Zone C (0.7).

**Table 2:** Reported^27,28^ and simulated infection risks overall, and for aisles seats only and non-aisle seats only.

Outbreak		Infection risk (%)			
		Chosen simulation^b^	Average of 100 simulations (95% CI)	Reported outbreak data (no. of infected/total susceptible)	
**GII 737** ^a^	Overall	8.3	8.0 ([7.4, 8.6])	8.5 (6/71)	
	Aisle seats	20.0	16.0 ([14.8, 17.1])	20.8% (5/24)	Relative risk: 9.5 95% CI: 1.2 to 77.4 P-value: 0.008
	Non-aisle seats	2.8	4.3 ([3.9, 4.9])	2.1% (1/47)	
**GI 747** ^c^	Overall	28.8	25.6 ([25.2, 26.1])	33.6 (41/122)	
	Aisle seats	36.6	35.8 ([35.1, 36.5])	37.2% (19/51)	Relative risk: 1.2 95% CI: 0.7 to 2.0 P-value: 0.47
	Non-aisle seats	24.0	19.2 ([18.7, 19.6])	31.0% (22/71)	

^a^Only non-tour group members in Economy class were focused on here, as the tour group members might have had interaction with the index patient(s) before and after the flight.

^b^This particular simulation was chosen because the predicted spatial distribution of the secondary cases (Fig. 4b) was close to the reported distribution of cases (Fig. 4c).

^c^Only Zones C and D were studied because these two zones were adjacent to the vomiting incident, and most secondary cases (82%) were in these two zones.

## Discussion

To our knowledge, this study is the first to identify the existence of a surface contamination network in a crowded environment. In such a network, each high-touch surface can be touched by many people, and each of them can touch other surfaces, and so the process goes on. In the beginning of the process, there is only a small number of contaminated hands, and hence the growth of the network is slow. As time passes, more contaminated hands touch uncontaminated surfaces, and rapid growth follows. The number of uncontaminated surfaces decreases towards the latter part of the process, and the growth again slows. Eventually, all surfaces are contaminated, as the total number of high-touch surfaces is limited. This is the logistic growth of the number of contaminated surfaces.

This finding has at least two significant implications. The first concerns the rapidity of the growth of the network, which suggests that the fomite route can be potentially fast and effective, as shown by the GII norovirus outbreak^25^. The existence of a surface touch network means that many surfaces can be contaminated. The community structure of the surface contamination network in an aircraft cabin is interesting. As the connection between different communities, the aisle seatback surfaces become the root surfaces for the surfaces within each sub-community. The aisle seatback contamination network has a small diameter (5), so all aisle seatback surfaces have the potential to become contaminated. Also, even remote surfaces can be contaminated, and the virus particles can travel as far as the contaminated hands do. This suggests that infection transmitted a great distance is not a unique feature of the airborne route as is commonly believed. In a number of studies, remote infection was considered to be direct evidence for airborne transmission^33^. Marks *et al*.^34^ found an inverse relationship between the attack rates at dining tables and the distance from the person who vomited, and concluded that the transmission might be airborne. Our results here suggest that the evidence provided by Marks *et al*.^34^ may not be adequate, because it is possible for infection risk to show an inverse relationship with the distance from the infector even when transmission is only via the fomite route, as shown in the norovirus outbreak simulation (Fig. 4a).

Different aisle seatback surfaces experience different contact rates during a flight. A high contact rate often corresponds to a high concentration of virus particles on a surface. This agrees with some field studies showing that high-touch surfaces may be a reservoir for nosocomial pathogens^13^. High-touch surfaces play a more important role in the fomite route. The hands of healthcare workers are likely to be contaminated with pathogens after contact with high-touch surfaces, similar to direct contact with patients^35^. The US Centers for Disease Control recommends cleaning and disinfecting high-touch surfaces on a more frequent basis than minimal-touch surfaces in hospitals^36^. A field study by Bogusz *et al*.^37^ qualitatively showed that hospital surfaces can be rapidly contaminated after cleaning, and high-touch surfaces have a higher frequency of contamination than other sites. From the surface cleaning perspective, a study by Kundrapu *et al*.^5^ showed that daily disinfection of high-touch surfaces can reduce acquisition of pathogens on hands. Disinfection should focus on high-touch objects and surfaces rather than on all surfaces^13^. The aisle seatback surfaces are relatively high-touch surfaces. Each seatback can be touched by the passenger sitting behind, which leads to the aisle seat passengers having a higher infection risk than others. This implies that when implementing inflight infection control measures for fomite transmission, high frequency disinfection should be applied not only to toilets, but also to aisle seatback surfaces, especially those close to toilets.

The models we have developed may be useful for improving the existing mathematical models for the fomite route transmission of influenza or the norovirus^21,22,32^. The existing models do not consider the surface touch network, so they cannot reveal the different roles that high- and low-touch surfaces play.

The major limitation of this study lies in the assumptions about surface touch behaviour, due to a lack of data. The agreement between our model and data from the two reported outbreaks was surprisingly good, suggesting that our assumptions about surface touch behaviour are plausible. The predicted infection risk depends on a large number of parameters. The most important parameters are the virus particle source strength, dose-response parameters, surface transfer rates, and nominal contact surface areas. The choice of some of these parameters has yet to be properly validated. Further research into determining the choice of parameter is needed.

## Methods Summary

In this study, we firstly used ordinary differential equations (ODEs) to analyse the growth of the number of contaminated surfaces in an idealized situation that all individuals touch all surfaces homogeneously. Then we performed a computer simulation of surface contamination process in the real air cabin environment, using random discrete-time Markov Chain approach. And to verify our model, we also simulated two inflight norovirus outbreaks. At last, we did a bench-top experiment to explore growth of contaminated surfaces in the same air cabin environment as the computer simulation.

### Theoretical study of the growth of contaminated surfaces

We used ordinary differential equations (ODEs) to analyse the relationship between the growth of the number of surfaces contaminated with live pathogens, an individual’s surface touching behaviour, and hand and surface hygiene. We built the model in an enclosed environment based on the assumption that all individuals touch surfaces homogeneously. Then we can write the following governing equations for the percentage of contaminated surfaces (*y*_1_(*t*)) and individuals with contaminated hands (*y*_2_(*t*)) respectively.

1 $$\begin{array}{c}\frac{d{y}_{1}(t)}{dt}={c}_{p}{y}_{2}(t)(1-{y}_{1}(t))-{d}_{p}{y}_{1}(t)\\ \frac{d{y}_{2}(t)}{dt}={c}_{s}{y}_{1}(t)(1-{y}_{2}(t))-{d}_{s}{y}_{2}(t)\end{array}$$

Where *c*_*p*_ is the hand contact frequency on environmental surfaces, which is defined as the total number of surface-to-hand contacts per time unit divided by the number of people; *c*_*s*_ is the surface contact frequency, which is defined as the total number of surface-to-hand contacts per time unit divided by the number of surfaces; *d*_*p*_ and *d*_*s*_ are the hand and surface hygiene frequency (See detailed construction of the ODEs in Supplementary Information Section 1.2).

### Computer simulation of surface contamination network in air cabin

We also constructed a surface contamination network during the flight using random discrete-time Markov chain method. We use the sub-group based approach, i.e., dividing all surfaces *N*_*s*_ and population *N*_*p*_ into sub-groups (e.g., toilet surfaces *N*_*s*1_, seat backs *N*_*s*2_, armrest surfaces, *N*_*s*3_, tray table surfaces *N*_*s*4_) and population sub-groups (e.g., passengers *N*_*p*1_, air crews *N*_*p*2_) and model events of surface touch, i.e., how each population sub-group *j* interacts with surface sub-group *i*. Five commonly observed surface touching behaviours during the cruise phase are considered in this study: toilet use, touching the aisle seat backrest surfaces on the way to the toilets and back, touching the armrest surface, touching the front backrest surface and touching the tray table surfaces. We impose different assumptions in each sub-group based on the nature of interaction, which is determined by human behaviour and nature of the activity, e.g., people go to the toilet following the principle of proximity. The time-dependent surface touch patterns can be easily considered in our model, e.g., visiting toilets. The frequency of surface touching by each sub-group of people on each sub-group of surfaces is assumed to be deterministic, however, some randomization exists in the touch sequence and exact timing of each touch. (See details in Supplementary Information Section 1.4).

**Figure 5:**
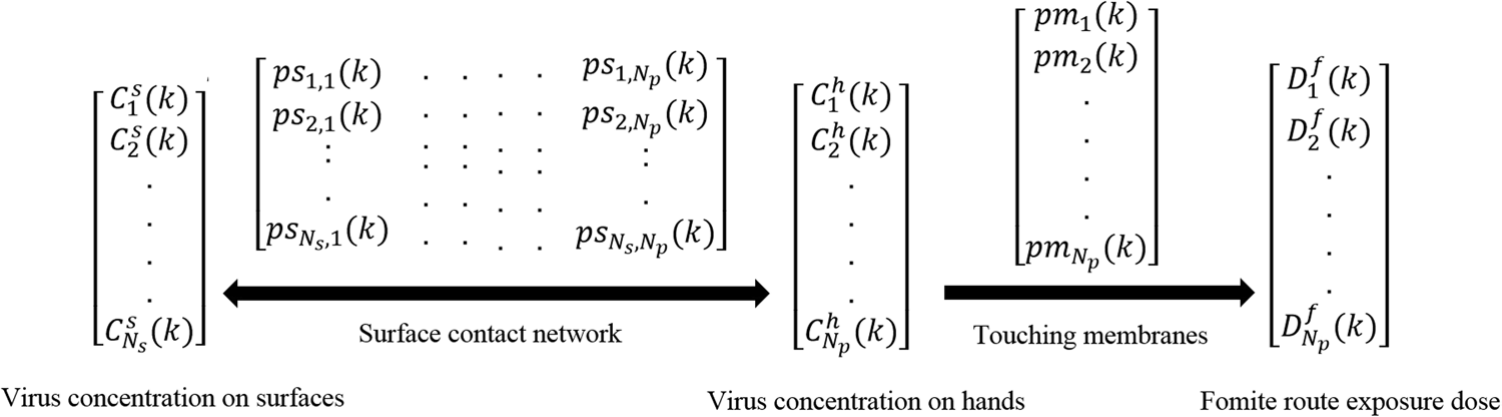
Diagram of the surface contact network and fomite route exposure model.

The diagram of the surface contact network and fomite route exposure model is shown in Fig. 5. The time step Δ*T* is an important parameter. It should be sufficiently small so that a surface cannot be touched by more than one individual at the same time, but not too small, to save on computation time. It depends on the crowdedness of the environment (See details in Supplementary Information Section 1.4). Denote virus concentration on surface *j* and hands of individual *i* at time step *k* be *C* ^*s*^_*j*_(*k*) and *C* ^*h*^_*i*_(*k*) respectively. At the next time step *k* + 1 after time Δ*T*, the virus concentration on the contact area is

2 $${C}_{j}^{s}(k+1)=(1-{r}_{s})({C}_{j}^{s}(k)+\frac{{\sum }_{i=1}^{{N}_{p}}({C}_{i}^{h}(k){A}_{h}{\tau }_{h{s}_{j}}-{C}_{j}^{s}(k){A}_{h}{\tau }_{{s}_{j}h})p{s}_{i,j}(k)}{{A}_{{s}_{j}}}){e}^{-{b}_{{s}_{j}}\times {\rm{\Delta }}T}$$

Where *r*_*s*_ is the removal rate of virus/particles by surface hygiene at time *K* + 1 if episodic surface cleaning/disinfection is carried out. ${b}_{{s}_{j}}$ is the virus first-order inactivation rate on surface *j*. ${\tau }_{h{s}_{j}}$ and ${\tau }_{{s}_{j}h}$ are the virus/particle transfer efficiency from hand to surface *j* and surface *j* to hand respectively. *A*_*h*_ and ${A}_{{s}_{j}}$ are the hand palm and surface *j* area.

To calculate the number of infective pathogens on hands, we considered the virus transfer from hands to mucous membranes during eye-rubbing, mouth-touching and nose-blowing. We assume that this process is a one-way transmission, i.e., from hands to mucous membranes. Denote *pm*_*i*_(*k*) as the mucous membrane touching behaviour of individual *i*, i.e., if at time interval *k*, individual *i* touches his or her mucous membranes, *pm*_*i*_(*k*) = 1, otherwise *pm*_*i*_(*k*) = 0. We assume that touching a mucous membrane involves only one fingertip with contact area *A*_*hm*_. Then the virus concentration on the hand of individual *i* at time step *k* + 1 becomes

3 $$\begin{array}{ccc}{C}_{i}^{h}(k+1) & = & (1-{r}_{h})({C}_{i}^{h}(k)-\frac{{\sum }_{j=1}^{{N}_{s}}({C}_{i}^{h}(k){A}_{h}{\tau }_{h{s}_{j}}-{C}_{j}^{s}(k){A}_{h}{\tau }_{{s}_{j}h})p{s}_{i,j}(k)}{{A}_{h}}\\  &  & -\frac{\,p{m}_{i}(k){A}_{hm}{\tau }_{hm}{C}_{i}^{h}(k)}{{A}_{h}})\,{e}^{-{b}_{h}\times {\rm{\Delta }}T}\end{array}$$

Where *r*_*h*_ is the removal rate of virus/particles by hand hygiene at time *K* + 1 if hand hygiene is carried out. *b*_*h*_ is the first-order inactivation rate of the virus on hands, *τ*_*hm*_ is the virus transfer rate from the hand to mucous membranes.

Given the virus particle concentrations on all surfaces and hands at the initial condition (k = 0), then the virus concentrations at each time step can be calculated as above.

The total exposure dose of individual *i* via the fomite route becomes

4 $${D}_{i}^{f}=\sum _{k=1}^{\,{N}_{t}}\,p{m}_{i}(k){A}_{hm}{\tau }_{hm}{C}_{i}^{h}(k)$$

The dose-response model is used to calculate infection risk. At the exposure dose of *D* ^*f*^_*i*_, both via the airborne route and the fomite route, the infection risk is $\,P=1-{e}^{-\eta {D}_{i}^{f}}$, where $\eta \,\,$is the dose-response rate.

The basic theory of networks^38^ was used to analyse the following properties of the network: the maximum vertex (highest-touch surface, ranking vertices), the maximum edge (the highest-touch people, ranking edges), the maximum geodesic distance (diameter), the average geodesic distance and the average clustering coefficient. The maximum geodesic distance (diameter) is the number of generations required for surfaces to be contaminated.

Note that surfaces in the environment can also acquire viruses through the deposition of small droplets in the air, and direct shedding from patients, which can be estimated based on existing approaches^39^. We used our model to evaluate the models developed against two reported inflight norovirus outbreaks^25,26^. The aircraft cabin is a special environment with fixed passengers and crew members, and people cannot move about as easily as in other settings. The unique feature of our model is its ability to predict the spatial distribution of infection risks. The model involves a large number of parameters. The chosen parameter values are listed in Supplementary Information Table S5, along with the references. The sensitivity of key parameters was studied and is reported in the Supplementary Information Section 2.1. For each parameter setting, we performed 100 random tests for each simulation to compare the total exposure dose for all susceptible individuals, as many processes are random. The parameters varied included the frequency of touching mucous membranes and other environmental parameters.

### Bench-top experiment study of surface contamination networks

**Figure 6:**
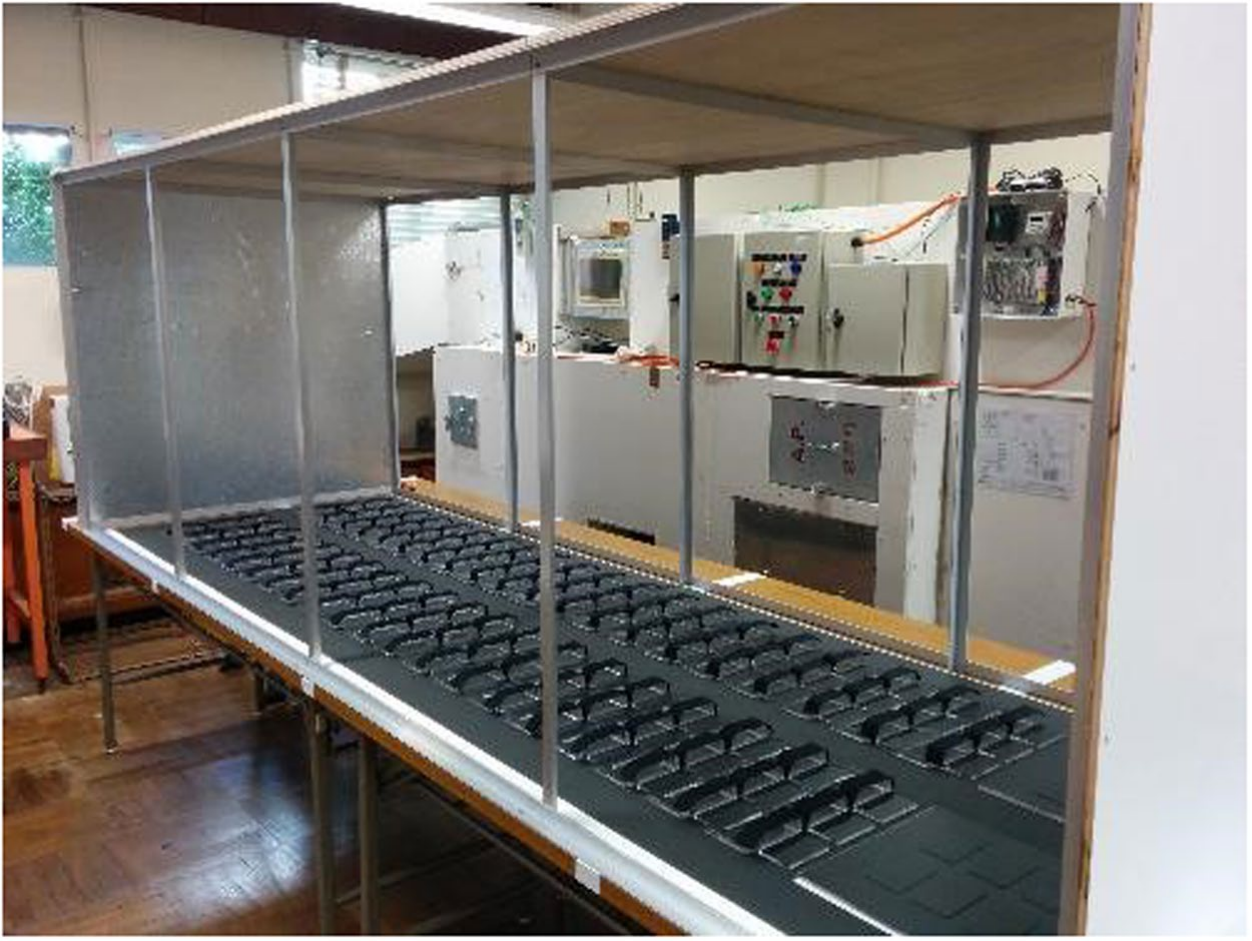
The laboratory table set up for the surface contamination network studies.

To study growth in the number of contaminated surfaces, we also built a bench-top experiment using a mock-up of an aircraft cabin, as shown in Fig. 6. Participants were instructed to touch the surfaces (chips) in a specific sequence, and/or use a specific type of grasp (light touch for the results shown here, firm touch, sliding, etc.). The computer-generated sequence follows the same rules as in the computational simulations. Qualitative observation of fluorescence using UV lamps indicated how many chips/fingers were contaminated at different times, showing how the fluorescent particles were transferred from the initial contaminated surface over eight (or some other number of) consecutive surfaces (see details in Supplementary Information Section 1.3).

### Statistics

Two-side Chi-Square test was used to test whether the aisle passengers had a statistically significant higher infection risk than non-aisle passengers from outbreak data. The ***P*** value < 0.05 was considered significant. And 95% confidence interval of the relative risk (aisle passengers to non-aisle passengers) was also provided.

### Data availability

The datasets generated during and/or analysed during the current study are available from the corresponding author on reasonable request.

## Electronic supplementary material

Supplemental Information
