# Supplementary material for: Logistic growth of a surface contamination network and its role in disease spread
Source: Sci Rep. 2017 Nov 1;7:14826. doi: 10.1038/s41598-017-13840-z (PMC5665872; doi:10.1038/s41598-017-13840-z)
Supplement: Supplementary file 1 — Supplemental Information [file 41598_2017_13840_MOESM1_ESM.pdf]

SUPPLEMENTAL INFORMATION

Logistic growth of a surface contamination network and its role in disease spread

1. SUPPLEMENTARY METHODS

1.1 The spatial distribution of passengers in the two outbreaks

The detailed environmental setting and spatial distribution of passengers in the two outbreaks are shown in Figure S1.

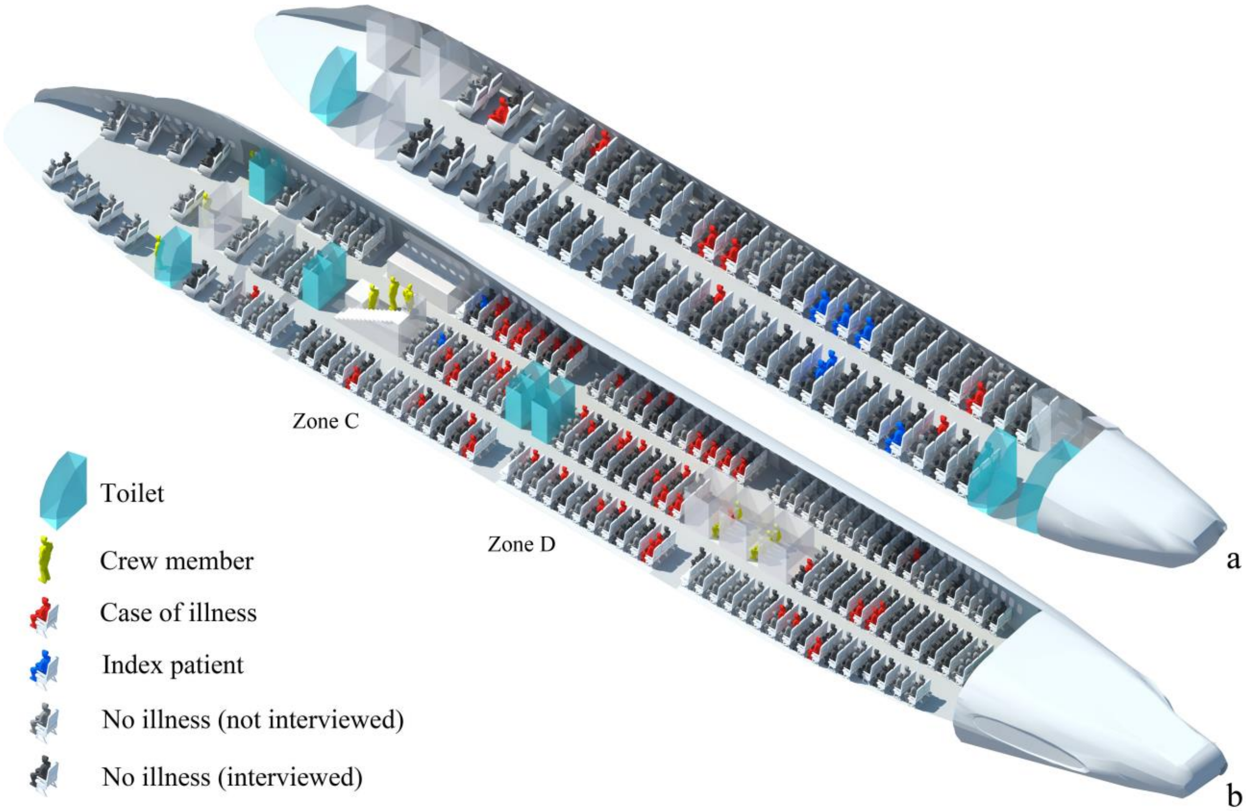

Figure S1 The reported spatial distribution of gastrointestinal cases on two flights, (a) Norovirus GII 737[1], (b) Norovirus GI 747[2] . Zones C and D in the 747 cabin are highlighted.

1.2 Theoretical study of the growth of contaminated surfaces

We proposed a mathematical approach using ordinary differential equations (ODEs) to analyse the relationship between the growth of the number of surfaces contaminated with live pathogens, an individual's surface touching behaviour, and hand and surface hygiene. We built the model in an enclosed environment based on the following assumptions:

- The total number of surfaces N_s is constant. $N_s = N_{sd}(t) + N_{sc}(t)$, where $N_{sd}(t)$ and $N_{sc}(t)$ are the number of dirty (contaminated with live pathogens) and clean surfaces at time t respectively.
- The total population size N_p is constant. $N_p = N_{pd}(t) + N_{pc}(t)$, where $N_{pd}(t)$ and $N_{pc}(t)$ are the numbers of individuals with dirty (contaminated with live pathogens) and clean hands at time t respectively.
- Populations touch portions of the fomite homogeneously.
- Cleaning of surfaces and hands occurs uniformly. For example, if the surface cleaning rate is two times per hour, all surfaces would be disinfected every half hour, and the disinfection efficacy would be 100%.

Some parameters used in the model are defined in Table S1.

Table S1 Parameters in the equations.

Parameter	Description
c_s	Surface contact rate, the total number of surface-to-hand contacts per unit of time divided by the number of surfaces.
c_p	Hand contact rate, the total number of surface-to-hand contacts per unit of time divided by the number of people. $c_s N_s = c_p N_p$
d_p	Pathogen cleaning rate of individual hands.
d_s	Pathogen cleaning rate of surfaces.

In each time unit, $c_p \times N_p$ hand-to-surface touching behaviours occur, and only a clean hand touching a dirty surface can lead to an increased number of dirty hands. Based on the assumption that populations touch fomites homogeneously, $c_p \times N_p \times \frac{N_{sd}(t)}{N_s} \times \frac{N_{pc}(t)}{N_p} = c_p N_{pc}(t) \times \frac{N_{sd}(t)}{N_s}$ clean hands-to-dirty surfaces touching behaviours occur in each time unit. Thus,

$$\frac{dN_{pd}(t)}{dt} = c_p N_{pc}(t) \frac{N_{sd}(t)}{N_s} - d_p N_{pd}(t) \qquad (1)$$

Similarly,

$$\frac{dN_{sd}(t)}{dt} = c_s N_{sc}(t) \frac{N_{pd}(t)}{N_p} - d_s N_{sd}(t) \qquad (2)$$

There is no analytic solution for Equations (1) and (2), so the dynamic properties and numerical solutions for different situations are obtained as follows:

The dynamic properties of Equations (1) and (2) indicate that

when $c_p c_s - d_p d_s \geq 0$, $N_{pd}(\mathrm{t}) \to \frac{N_p(c_p c_s - d_p d_s)}{c_p(c_s + d_s)}, N_{sd}(\mathrm{t}) \to \frac{N_s(c_p c_s - d_p d_s)}{c_s(c_p + d_p)}$, , as $t \to \infty$,
and $c_p c_s - d_p d_s < 0$, $N_{pd}(\mathrm{t}) \to 0, N_{sd}(\mathrm{t}) \to 0$, as $t \to \infty$.

Suppose in an enclosed environment there are 100 non-porous surfaces and 100 individuals, and on average every individual touches one surface every 30 minutes, then $c_p = 0.033/\mathrm{min}$. $c_s = (100 \times 2)/(100 \times 60) = 0.033/\mathrm{min}$.

Take the influenza virus as an example. The influenza virus can survive for 24–48 hours on hard and non-porous surfaces[3]. On hands (skin), after being incubated on the skin for an hour, only 1% of the parainfluenza virus survives[4]. If there is no hand hygiene or disinfection of surfaces, taking the average value for hard, non-porous surfaces, $d_s = 1/36\ \text{hr} = 4.6 \times 10^{-4}$/min; for hands $d_p = 1.7 \times 10^{-2}$/min.

When surface cleaning frequency is low, such as once every 6 hours, and all individuals wash and clean their hands once every 2 hours, then $d_s = (1/(6 \times 60) + 4.6 \times 10^{-4})/\text{min} = 0.0032/\text{min}$ and $d_p = (1/(2 \times 60) + 1.7 \times 10^{-2})/\text{min} = 0.025/\text{min}$. Then $c_p c_s - d_p d_s > 0$. The numerical solutions of Equation (1) and (2) with the initial condition $y_{sd}(0) = 0$, $y_{pd}(0) = 1$ are shown in Figure 3e.

When surface cleaning frequency is high, i.e., once every half an hour, and all individuals clean their hands every 30 minutes, then $d_s = (1/30 + 4.6 \times 10^{-4})/\text{min} = 0.034/\text{min}$, $d_p = (1/30 + 1.7 \times 10^{-2})/\text{min} = 0.050/\text{min}$. Then $c_p c_s - d_p d_s < 0$. The numerical solutions of Equations (20) and (21) with the initial condition $y_{sd}(0) = 10$, $y_{pd}(0) = 0$ are shown in Figure S2.

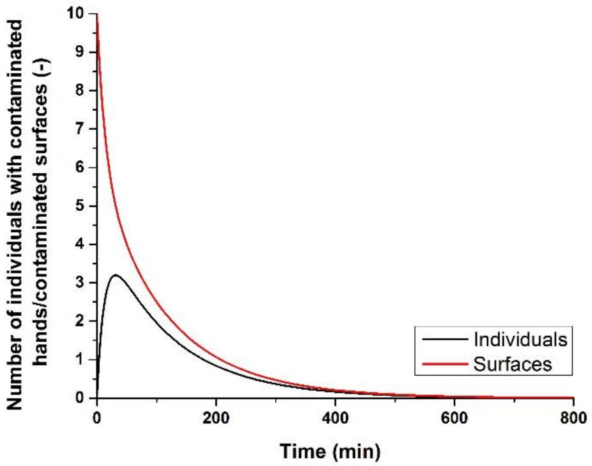

Figure S2. Numerical solutions for a high surface/hand hygiene rate.

1.3 Bench-top experiment study of surface contamination networks

To study growth in the number of contaminated surfaces, we also built a bench-top experiment using a mock-up of an aircraft cabin, as shown in Figure 6 in the main text. As in our computer simulations, four kinds of surface were considered, namely 120 seatbacks, 160 armrests, 120 tray tables and 2 toilets (refined into outside and inside door handles, toilet seat covers, toilet buttons, taps, sanitisers and other surfaces). The sketch of the aircraft cabin was printed on a piece of waterproof paper (0.9 m by 3 m), and the height of the experiment chamber was 1 m. The surface materials were simply divided into two types according to differences in porosity, with Dacron applied on the seatbacks and a modified propylene polymer on the others. To better

simulate the touching process, the dimensions of surfaces in the experiment were reduced from those in the real air cabin by a proportion of 5.17, which is the ratio of palm size to finger size.

Participants were instructed to touch the surfaces (chips) in a specific sequence, and/or use a specific type of grasp (light touch for the results shown here, firm touch, sliding, etc.). The computer-generated sequence follows the same rules as in the computational simulations. Participants' fingerstalls stand for people in the aircraft cabin, i.e., passengers or air crew members. Either participants' fingers were smeared with fluorescent particles, or fluorescent particles were applied to specific chips, known only to the researchers. To reduce costs, one human subject could play up to ten roles if each finger represents one person. This simple design allowed us to complete one test in 5 to 10 hours. Qualitative observation of fluorescence using UV lamps indicated how many chips/fingers were contaminated at different times, showing how the fluorescent particles were transferred from the initial contaminated surface over eight (or some other number of) consecutive surfaces.

We made an assumption in the experiments that all surfaces were clean before the flight, which might not be true in reality. We considered that there were two index patients during the flight, seated in 8B and 9E. Figure S3a shows the contamination conditions of surfaces in the experiment at Round 15. The white dots shoe areas contaminated with fluorescent particles; brighter dots indicate a larger number of fluorescent particles. In the experiment, we took a photograph after a passenger used the toilet and went back to his or her seat (the entire process was considered to be one round).

To make the contamination of each surface more discernible, MATLAB 2013b was used to exclude the influence of reflected light and preserve the affected parts of the photo, namely the light emitted by fluorescent particles. The contamination conditions in Rounds 8, 15, 22, 29, 36, 43 are shown in Figure S3b–g.

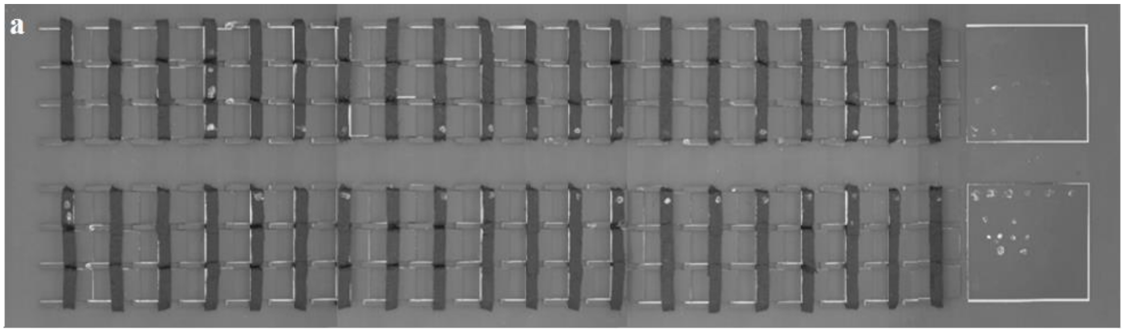

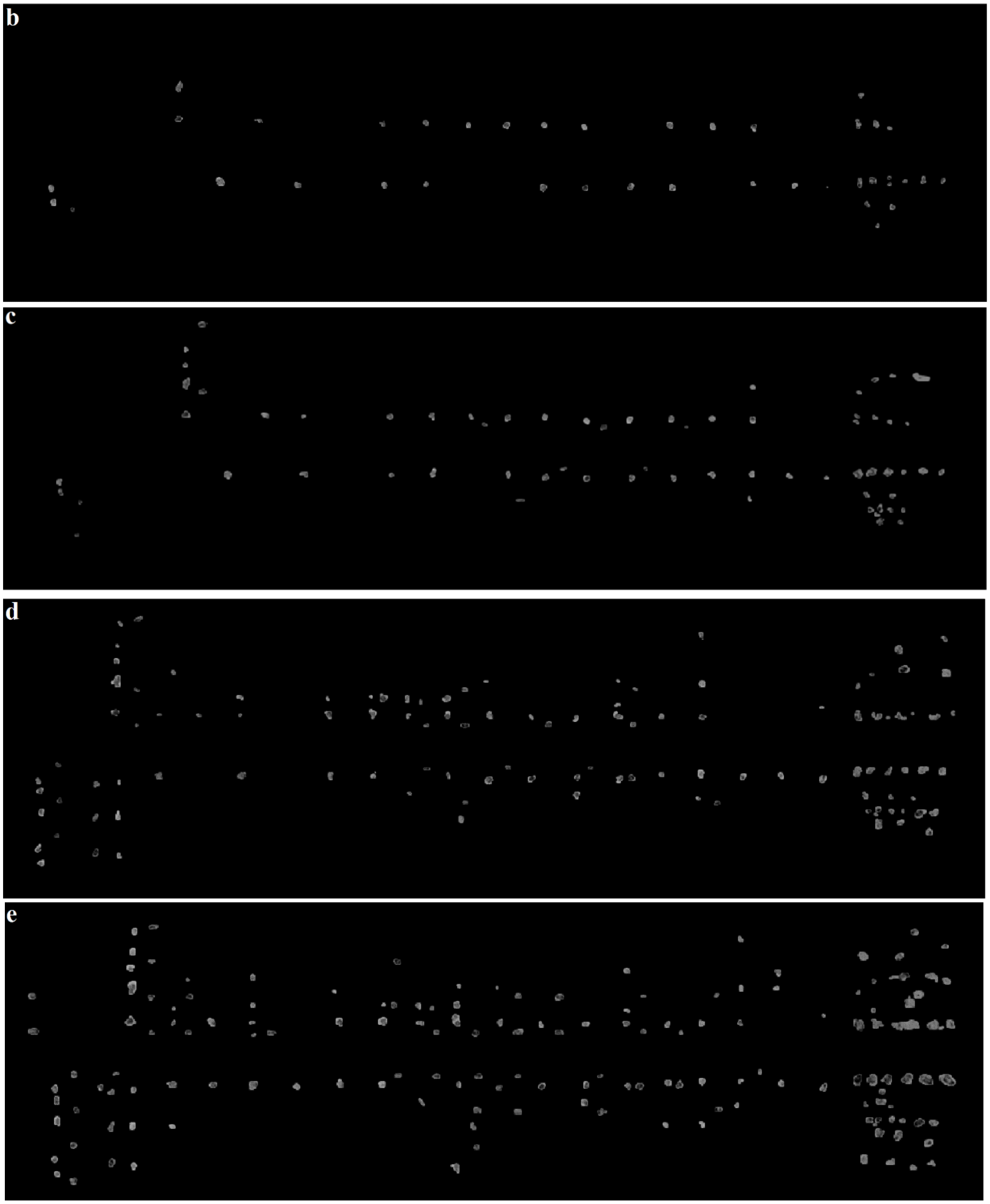
b
c
d
e

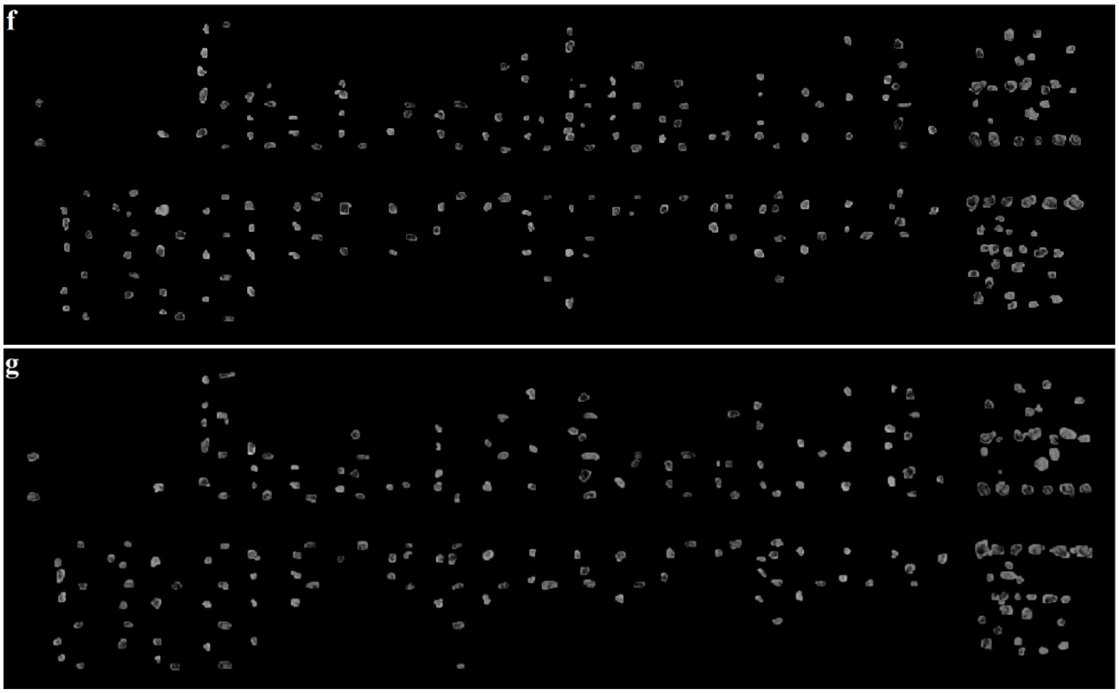

Figure S3. a) Contamination conditions of surfaces in one experiment, Round 15. Photos processed from different rounds: b) Round 8, c) Round 15, d) Round 22, e) Round 29, f) Round 36, g) Round 43.

Figure 3f in the main text shows the relationship between time length (represented by the number of rounds) and the number of contaminated seatback surfaces along the aisle. The number of contaminated seatback surfaces along the aisle first begins to increase quickly with time, and then slows. Therefore, a logistic curve was used to fit the data. The R-square and chi-square values of the logistic regression are 0.906 and 14.10 respectively, which means that the logistic curve is a good fit for the data.

1.4 Modelling the fomite route

We constructed a surface contamination network with the following assumptions. Individual differences in surface touching behaviour were ignored. For each type of surface, the touching behaviour for each individual was assumed to be the same and the interval between two subsequent touching behaviours was equal. A constant virus transfer rate was assumed for each type of surface. The surface transfer rates were obtained from existing studies of porous, non-porous and wet surfaces. We ignored the influence of touch pressure and other factors on transfer rate.

Constructing the surface contamination network

We built the surface contamination network in an air cabin environment with detailed flight information in Table S2.

Table S2 Flight information parameters.

Parameter	Description
T	Flight duration
T_{ac}	Duration after the cruise phase and before the end of the flight
N_{pe}	Number of passengers in the flight
N_{ce}	Number of crew member in the flight
N_{ie}	Number of infectious cases in the flight

During the taxi-out and climb phases of a flight, there is no movement of passengers, so surface virus accumulations are due to the deposition of the airborne viruses alone. There is currently a lack of data on surface touching behaviour. We considered five commonly observed surface touching behaviours during the cruise phase: toilet use, touching the aisle seat backrest surfaces on the way to the toilets and back, touching the armrest surface, touching the front backrest surface and touching the tray table surfaces. And the frequency of these five surface touching behaviors are listed in Table S3.

Table S3 Frequency of the modelled five surface touching behaviors during air travel.

Parameter	Description	Value	Source
f_{st}	Toilet use frequency for susceptible individual [1/hr]	1/6	Assumed
f_{it}	Toilet use frequency for infector [1/hr]	1/3	Assumed
P_{tasb}	Probability that an individual will touch certain aisle seatback surfaces on the way to toilets and back [-]	1/6	Assumed
$f_h^{i,as}$	Frequency for individual i to touch the armrest surfaces [1/hr]	5	Assumed
$f_h^{i,sb}$	Frequency for individual i to touch the immediate front seatback surfaces [1/hr]	3	Assumed
$f_h^{i,tt}$	Frequency for individual i to touch the tray table surfaces [1/hr]	4	Assumed

To specify behaviour patterns, we divided the cruise phase into several time intervals. In each time interval, the above surface touching behaviours of each individual can occur once at most. We placed all behaviours at different time intervals based on certain mechanisms. Then similar to the study by King *et al*[5], a Markov Chain was built to calculate the exchange of viruses between surfaces and hands at each time step, and also the fomite route exposure. The smaller the time interval, the closer the solution will be to the actual situation, but a finer division also means more calculations. The duration between two sequential surface touching for five kinds of

touching behaviours is listed in Table S4. Generally, due to the large number of passengers, the duration between two sequential toilet visit ΔT_{te} is always much smaller than the duration between two sequential seatback surface touching, armrest surface touching, tray table surface touching, and mucous membranes touching, so we divided the cruise phase by $\Delta T_{te}/2$, giving a total of $\frac{2T_c}{\Delta T_{te}}$ time intervals.

Table S4 Duration between two sequential same surface touching.

Surface touching behaviour	Duration between two sequential contacts
Toilet visits	$\Delta T_{te} = T_c \Big/ \left(\lfloor N_{ie} \times (T - T_{ac}) \times f_{it} \rfloor + \lfloor (N_{pe} + N_{ce} - N_{ie}) \times (T - T_{ac}) \times f_{st} \rfloor\right)$
Seatback surface touching	$1 \big/ f_h^{i,sb}$
Armrest surface touching	$f_h^{i,as}$
Tray table surface touching	$1 \big/ f_h^{i,tt}$
Mucous membranes touching	$1 \big/ f_h^{i,m}$

where N_{ite} and N_{ste} are the total number of toilet visits in Economy class for the infectors and susceptible individuals respectively, $\lfloor \quad \rfloor$ is the floor function. We assume the distribution of the total $N_{ite} + N_{ste}$ toilet visits in the entire cruise phase of the flight is uniform.

Next, we define when each type of behaviour occurs in the cruise phase.

First, the total number of toilet visits $(\lfloor N_{ie} \times (T - T_{ac}) \times f_{it} \rfloor + \lfloor (N_{pe} + N_{ce} - N_{ie}) \times (T - T_{ac}) \times f_{st} \rfloor)$ are uniformly distributed in the odd time steps during the cruise phase, and it was assumed that if a passenger uses the toilet at time step $2k - 1$, he or she will exit the toilet at time step $2k$ and return to his or her seat.

Second, we specified the aisle seatback surfaces that passengers would touch on the way to the toilet and back. We assumed the probability that a passenger would touch one aisle seat back surface in this situation is P_{tasb}, and there are s_j aisle seat back surfaces on the way to the toilet (s_j is determined by the passenger's seat number). Suppose the passenger sits in the jth row in Economy class. If using a toilet in the front, $s_j = 2j$; if using a toilet in the rear, $s_j = 2(N_{sre} - j)$. For each passenger who used the toilet, a probability from 0 to 1 was randomly assigned for all s_j aisle seatback surfaces. If the probability for one aisle seatback surface was smaller than P_{tasb}, we assumed that the passenger would touch this aisle seatback surface on the way to the toilet, otherwise he or she would not touch it. We similarly specified the aisle seatback surface that this passenger would touch on the way back from the toilet to his or her seat.

Third, we specified other four contact behaviours for each passenger: mucous membranes, front seatback surfaces, armrest surfaces and tray table surfaces touching. Take the mucous membranes touching as an example. The first time for individual i is randomly chosen, and then

after every $1/f_h^{i,m}$ time, the individual i would touch the mucous membranes once. The arrangements of front seatback surfaces, armrest surfaces and tray table surfaces touching are similar.

A surface contact network can be represented by the matrix $Ps = (ps(k))_{N_s \times N_p}$, which describes the probability of surface touching behaviours at time step k. N_p is the total number of individuals, and N_s is the total number of environmental surfaces. If at time step k, individual i touched surface j, $ps_{j,i}(k)$=1, otherwise, $ps_{j,i}(k) = 0$. The matrix Ps is the incidence matrix of the surface contamination network.

We built two matrices, $C_s(k)$ with a dimension of $(N_s \times \frac{2T_c}{\Delta T_{te}})$ and $C_h(k)$ with a dimension of$(N_{pe} + N_{ce}) \times \frac{2T_c}{\Delta T_{te}}$), where $C_{s_j}(k)$ represents the virus concentration on the jth surface at the end of time interval k, N_s is the total number of surfaces under consideration. $C_{h_i}(k)$ is the virus concentration on the individual i's hand at the end of time interval k. The diagram for the fomite route model is shown in Figure S4.

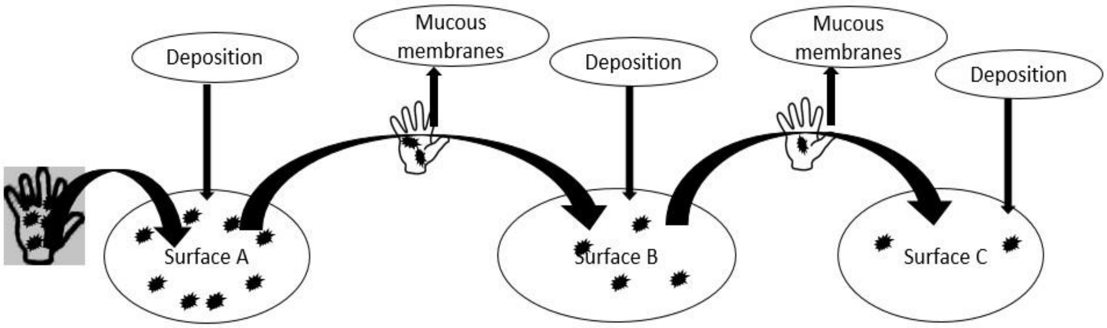

Figure S4. The fomite route model.

Initial virus concentration on surfaces

After vomiting, the mass of vomitus per hand is estimated to be 10^{-3} g**Error! Reference source not found.**, the virus concentration in faeces from a norovirus patient is assumed to be $L(r_0, 0)$ (genome/g), and the initial virus concentration on a patient's hand is $10^{-3}L(r_0, 0)/A_h$ (genome/m^2). If the patient vomited in the toilet, the concentration of faeces on the toilet surface is estimated to be 1g/cm^2 **Error! Reference source not found.**, if the toilet were cleaned after the patient vomited in it. Assuming the cleaning efficiency is η_c, then the initial virus concentration is (1-η_c) $L(r_0, 0)$ (genome/m^2).

Parameter selection

A large number of parameters are involved in our model. The chosen parameter values are listed in Table S5 along with the references. Parameter sensitivity studies were also carried out.

Table S5 Parameter values for the baseline case.

Parameter		Description		Value	Source
b_h		Norovirus first-order inactivation rate on hands [1/hr]		0.23	Estimated from Liu *et al.*[6]
b_{s_j}	b_p	Norovirus first-order inactivation rate on porous surfaces [1/hr]		0.1	Assumed
	b_{np}	Norovirus first-order inactivation rate on non-porous surfaces [1/hr]		0.095	Estimated from Cannon *et al.*[7]
	b_{wp}	Norovirus first-order inactivation rate on wet surfaces [1/hr]		0.027	Estimated from Cannon *et al.*[7]
$\tau_{s_j h_i}$	τ_{ph}	Transfer rate from porous surfaces to hands [-]		0.03	Sattar *et al.*[8]
	τ_{nph}	Transfer rate from non-porous surfaces to hands [-]		0.07	Mokhtari and Jaykus **Error! Reference source not found.**
	τ_{wh}	Transfer rate from wet surfaces to hands [-]		0.2	Mokhtari and Jaykus **Error! Reference source not found.**
$\tau_{h_i s_j}$	τ_{hp}	Transfer rate from hands to porous surfaces [-]		0.8	Estimated from Mackintosh and Hoffman[9]
	τ_{hnp}	Transfer rate from hands to non-porous surfaces [-]		0.12	Estimated from Lopez *et al.*[10]
	τ_{hw}	Transfer rate from hands to wet surfaces [-]		0.14	Mokhtari and Jaykus **Error! Reference source not found.**
τ_{hm}		Transfer rate from hands to mucous membranes [-]		0.35	Estimated from Rusin *et al.*[11]
A_{hm}		Hand contact area when touching mucous membranes [m^2]		0.0002	Nicas and Sun[12]
A_h		Contact area of hands and environmental surfaces [m^2]		0.013	Estimated
$f_h^{i,m}$		Frequency for individual i to touch his/her mucous membranes [1/hr]		5	Hendley *et al.*[13]
$L(r_0, t)$		Initial virus concentration in faeces from norovirus patient [cDNA genomes/g]	GI	8.4×10^5	Chan *et al.*[14]
			GII	3×10^8	
η_c		Toilet cleaning efficiency [-]		99%	Assumed

1.5 Modelling the airborne route

Faecal-oral spread is the primary mode of norovirus transmission[15]. However, it has been suggested that vomiting can produce aerosol droplets containing viral particles, which are inhaled by exposed susceptible individuals, deposited in the upper respiratory tract and then swallowed along with the respiratory mucus[16]. Airborne norovirus was detected in air samples from one norovirus outbreak in a healthcare facility[17]. A study of a hotel norovirus outbreak in which no food source was implicated found an inverse relationship between infection risk and the distance from the person who vomited[18]. These studies demonstrate the possibility of norovirus transmission via the airborne route. The potential for its fomite transmission is also supported by the widespread environmental contamination observed in a prolonged norovirus outbreak in a hotel[19]. Two previous investigations isolated norovirus RNA from environmental surfaces and suggested that environmental contamination is likely to have played a major role in prolonging the outbreaks[20]. In this study, we did not consider person-to-person contact because it is rare during a flight, especially between strangers; rather, we modelled the airborne route, as in the 747 GI outbreak, when the index patient vomited on the aisle.

Definition of the airborne transmission route

The airborne route refers to direct inhalation of an infectious agent through small droplet nuclei (the residue of large droplets containing microorganisms that have evaporated to a respirable aerodynamic radius of less than 5 μm)[21]. We separated airborne infection into short-range airborne infection (within 2 m of the index patient(s)) and long-range airborne infection (sharing the same indoor environment). In simulating the long-range airborne transmission, the cabin air flow is assumed to be fully mixed and the steady-state concentration of infectious agents can be quickly reached when the source release is constant.

In the 747 GI outbreak, the index patient vomited in the aisle. Microbiological data show that projectile vomiting associated with norovirus infection may distribute up to 3×10^7 virus particles as an aerosol with a total volume about 30 ml[22]. It is difficult for large droplets with a diameter greater than 10 μm to move as high as 1 m in the vertical direction, meaning they are unlikely to be inhaled by susceptible individuals. So for the airborne route of norovirus transmission, we considered only droplet nuclei with a diameter of less than 10 μm, which can be suspended in the air for a prolonged period.

Modelling the airborne route

The exposure dose in the upper respiratory tract of individual i due to the airborne route is denoted as D_{au}^{i}, and can be estimated as follows for flight duration T:

$$D_{au}^{i} = \int_{o}^{T} \int_{0}^{r_a} C_i(r,t) p \delta_u(r) t \frac{4}{3} \pi r_0^3 L(r_0,t) dr\, dt \quad (3)$$

where r_a is the largest radius for airborne droplets and $r_a = 5$ μm (Nicas and Jones, 2009); $C_i(r,t)$ is the concentration of the droplet with diameter r in the inhaled air of individual i; p is the pulmonary ventilation rate and $p = 0.48\ \mathrm{m}^3/\mathrm{hr}$ [23]; r_0 is the initial radius of the droplet, r is the final radius after complete evaporation, assuming that $r = r_0/3$ (Liu *et al.*); $\delta_u(r)$ is the deposition rate of droplets with radius r in the upper respiratory tract (the model from ICRP[24] was used in this study); $L(r_0,t)$ is the concentration of viable viruses (genome copies/ml) in droplets with initial radius r_0 at the time t after being exhaled. $L(r_0,t)$ changes with the flight time t because of the natural death of viruses in air.

$C_i(r,t)$ varies at different distances from the index patient. Liu *et al.* (unpublished) and Nielsen *et al.*[25] compared the airborne droplet concentration at different distances from the patient. A concentration ratio $\varepsilon_c(s_i) = C_i(r,t)/C_{aw}(r,t)$ was defined, where $C_{aw}(r,t)$ is the droplet nuclei concentration in the air away from the index patient, s_i is the horizontal distance between susceptible individual i and the location of vomiting, then $C_i(r,t) = \varepsilon_c(s_i)C_{aw}(r,t)$. The results show that ε_c decreases almost linearly when the distance increases to between 0.5 and 1 m, and at 1 m or more away from the index patient, ε_c fluctuates around unity. A simple model is used to describe the concentration ratio at distance s away from the patient, which is given in Equation (17).

$$\varepsilon_c(s) = \begin{cases} -6s+7, s<1 \\ 1, s \geq 1 \end{cases} \quad (4)$$

The rapid initial death rate of pathogens atomised into the air has been observed in many studies[26,27]. Evaporation of droplets is believed to play an important role[28]. Xie *et al.*[29] found that there is a stage of rapid decline in viability as droplets become completely evaporated, at which point viability decreases to about 25%, and then slowly declines. For airborne droplets with a diameter of less than 10 μm, the evaporation time is very short (less than 0.1 second[30]), so in this airborne transmission model, it is assumed that all the airborne droplets have been completely evaporated before being inhaled by susceptible individuals, and that the viable virus concentration is 25% of its initial value.

It is assumed that these droplets are uniformly and rapidly distributed in the air, which is set as the initial condition. Then the concentration of the norovirus-containing airborne droplets in the aircraft cabin can be calculated according to the following ordinary differential equation:

$$V\frac{\mathrm{d}(\varepsilon_c(s_i)C_{aw}(r,t)L(r_0,t))}{\mathrm{d}t} = -(q+b_a+kr^2)VC_{aw}(r,t)L(r_0,t) + (1-F_{HEPA})R_{re}qV\,C_{aw}(r,t)L(r_0,t)$$
$$C_{aw}(r,t_0) = 3\times10^7F_c(r_0)/V, \text{ for } r<5\ \mu\text{m} \quad (5)$$

where q is the air change rate (ACH) in the aircraft cabin and $q = 25/\text{hr.}$[31]; b_a is the first-order inactivation rate of virus in the droplet nuclei in the air. Due to the absence of data about the norovirus inactivation rate in air, it is represented by the influenza virus inactivation rate in air, $b_a = 0.22/\text{hr}$[32]. Due to the high air change rate in the aircraft cabin, q is much larger than b_a, so the result is not sensitive to small changes in b_a; kr^2 is the settling rate of the droplets on a surface with radius r, and $k = 0.1375/(hr.\ \mu\text{m}^2)$ (estimated from Thatcher *et al.*[33]); V is the volume of the aircraft cabin; F_{HEPA} is the efficiency of the HEPA filter and $F_{HEPA} = 99.97\%$; and R_{re} is the recirculation rate of the aircraft cabin ventilation system and $R_{re} = 0.5$.; and t_0 is the time when vomiting occurs.

$F_c(r_0)$ is the size distribution of the droplets from vomiting on the ground. Due to the absence of studies on this, the droplet size distribution from coughing in the study by Atkinson and Wein[34], which is given in Equation (19), is used.

$$F_c(r_0) = \begin{cases} \frac{0.71f_1(2r_0)+0.29f_2(2r_0)}{\int_0^{2000}(0.71f_1(2r)+0.29f_2(2r))dr} & for\ r_0 \leq 2000\ \mu\text{m} \\ 0 & for\ r_0 > 2000\ \mu\text{m} \end{cases} \tag{6}$$

where $f_1(x)$ and $f_2(x)$ are two lognormal distributions with geometric mean and geometric standard deviation 9.8 μm and 9 μm, and 160 μm and 1.7 μm, respectively.

The solution of Equation (18) is

$$C_{aw}(r,t)L(r_0,t) = C_{aw}(r,t)e^{-(q+b_a+kr^2-(1-F_{HEPA})qR_{re})(t-t_0)} \tag{7}$$

Infection risk assessment

The dose-response model is used to calculate infection risk. At the exposure dose of D, both via the airborne route and the fomite route, the infection risk is $P(= 1 - e^{-\eta D})$, where η is the dose-response rate. Human susceptibility to the norovirus is determined by secretor status (Se+/-)[35]. The susceptible individuals are divided into two groups: Se+ and Se- subjects. The susceptibility of Se+ and Se- subjects to norovirus varies greatly. The dose-response rates of norovirus GI for Se+ and Se- subjects is 0.14/genome copy and 9×10^{-4}/genome copy, respectively, and for norovirus GII it is 0.2 and 2.1×10^{-4}/genome copy for Se+ and Se- subjects, respectively[35]; it is assumed that 80% of the population are Se+ subjects and the rest are Se- subjects. The infection risk of a susceptible individual i becomes

$$P_i = 0.8 \times \left(1 - e^{-\eta_{mSe+}\cdot D^i}\right) + 0.2 \times (1 - e^{-\eta_{mSe-}\cdot D^i}) \tag{8}$$

2. SUPPLEMENTARY RESULTS

2.1 Sensitivity analysis of key parameters in computer simulations

The main limitations of our model lie in the assumptions about surface touching behaviour during the flight, due to a lack of data. Five commonly observed surface touching behaviours are taken into consideration: touching toilet surfaces (door handles, water faucets, toilet lids, and flushing buttons), touching aisle seat backrest surfaces on the way to the toilets and back, touching front backrest surfaces, touching armrest surfaces, and touching tray table surfaces.

To understand how human behavioural factors may affect the results of our analysis, we take the GII 737 aircraft cabin as an example. We examined the sensitivity of the average infection risk of all susceptible passengers, aisle seat and non-aisle seat passengers via the fomite route with various toilet usage frequencies, probabilities of touching one aisle seatback surface on the way to the toilets and back, frequency of touching front seatback surfaces, armrest surfaces, and tray table surfaces.

- We examined a range of toilet use frequencies for susceptible passengers from 1/12 to 1/2 per hour, as shown in Figure S5a. The baseline value of this parameter was 1/6 per hour.
- We examined a range of probabilities of touching one aisle seatback surface on the way to the toilets and back from 1/12 to 1/2, as shown in Figure S5b. In the baseline simulations, the value of this parameter was 1/6.

- We examined a range of frequencies of touching the front seatback surfaces for seated passengers from 1 to 9 per hour, as shown in Figure S5c. In the baseline simulations, the value of this parameter was 3 per hour.
- We examined a range of frequencies of touching armrest surfaces for seated passengers from 1 to 9 per hour, as shown in Figure S5d. In the baseline simulations, the value of this parameter was 5 per hour.
- We examined a range of frequencies of touching tray table surfaces for seated passengers from 1 to 8 per hour, as shown in Figure S5e. In the baseline simulations, the value of this parameter was 4 per hour

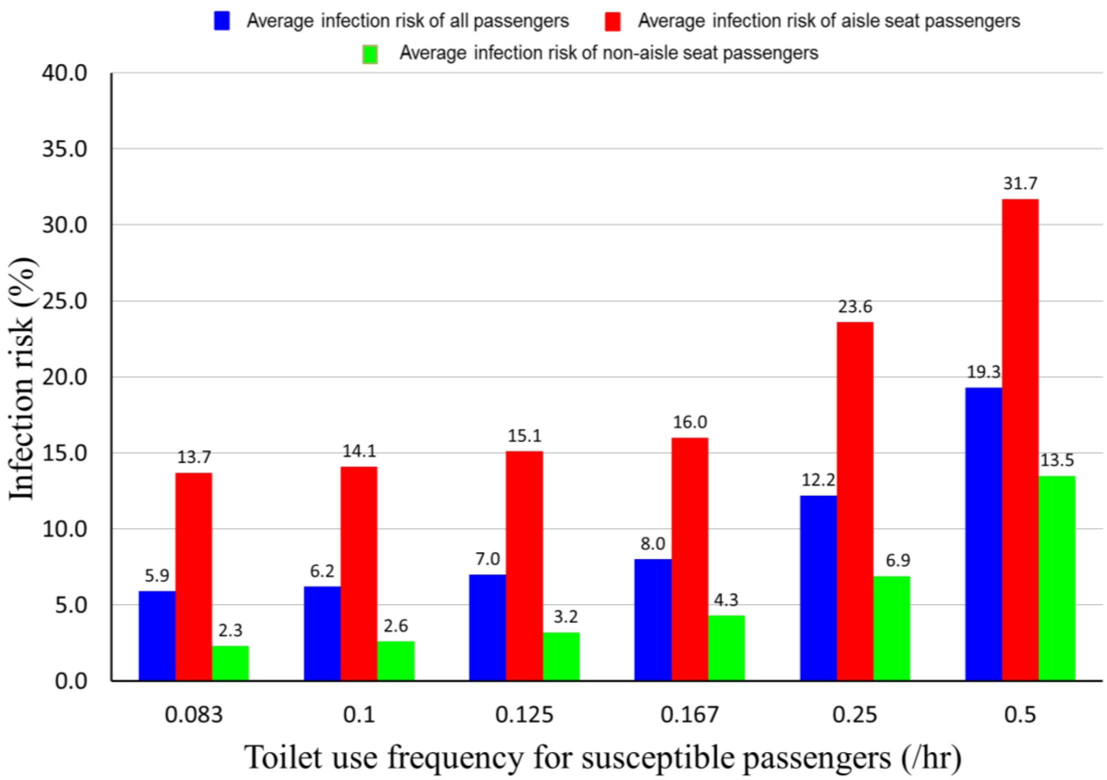

(a)

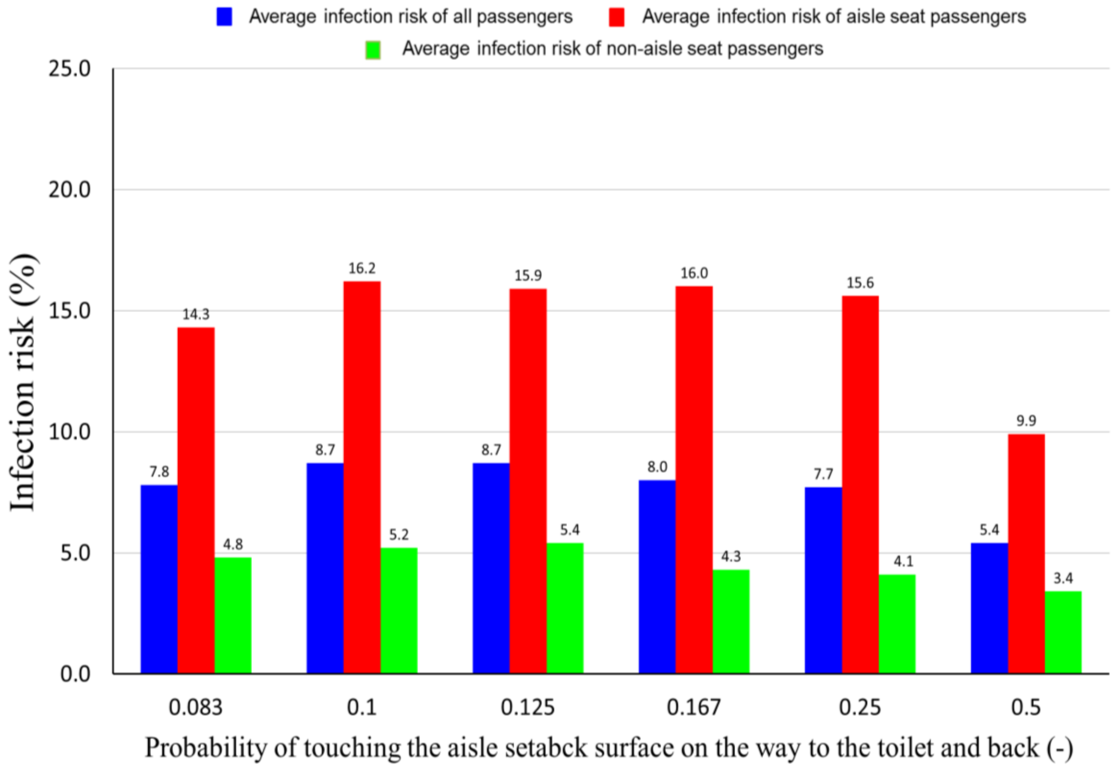
Average infection risk of all passengers
Average infection risk of aisle seat passengers
Average infection risk of non-aisle seat passengers
Infection risk (%)
25.0
20.0
15.0
10.0
5.0
0.0
7.8
14.3
4.8
8.7
16.2
5.2
8.7
15.9
5.4
8.0
16.0
4.3
7.7
15.6
4.1
5.4
9.9
3.4
0.083
0.1
0.125
0.167
0.25
0.5
Probability of touching the aisle setabck surface on the way to the toilet and back (-)

(b)

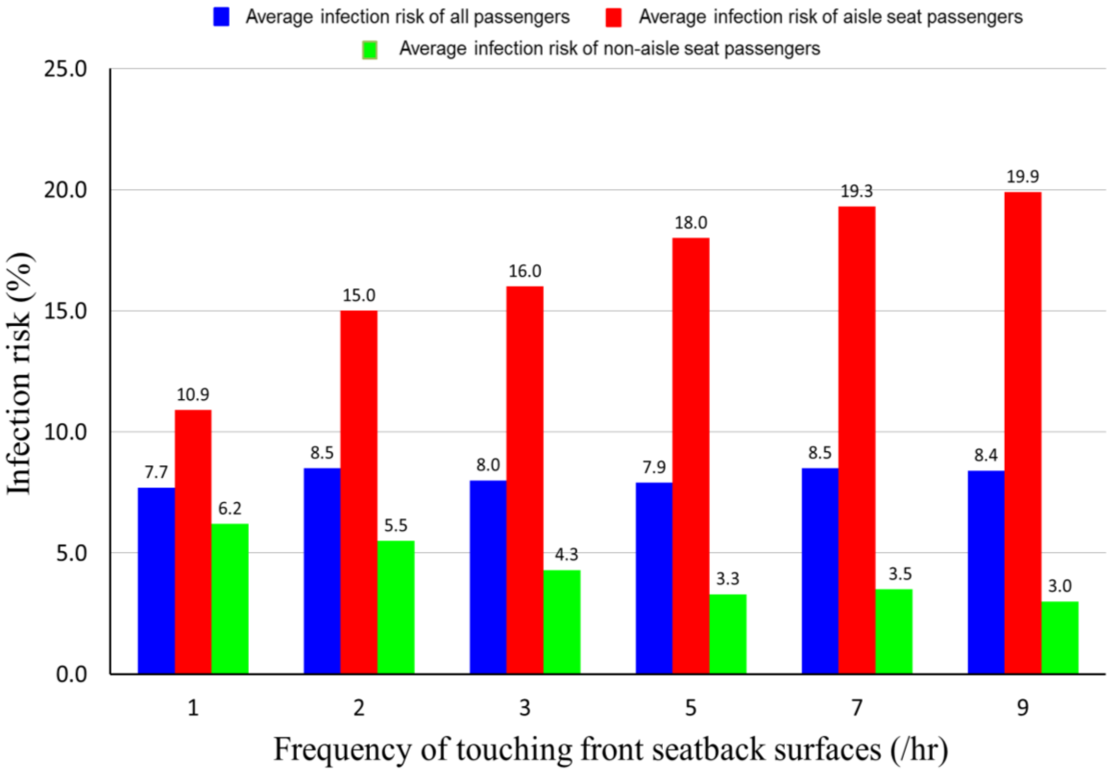
Average infection risk of all passengers
Average infection risk of aisle seat passengers
Average infection risk of non-aisle seat passengers
Infection risk (%)
25.0
20.0
15.0
10.0
5.0
0.0
7.7
10.9
6.2
8.5
15.0
5.5
8.0
16.0
4.3
7.9
18.0
3.3
8.5
19.3
3.5
8.4
19.9
3.0
1
2
3
5
7
9
Frequency of touching front seatback surfaces (/hr)

(c)

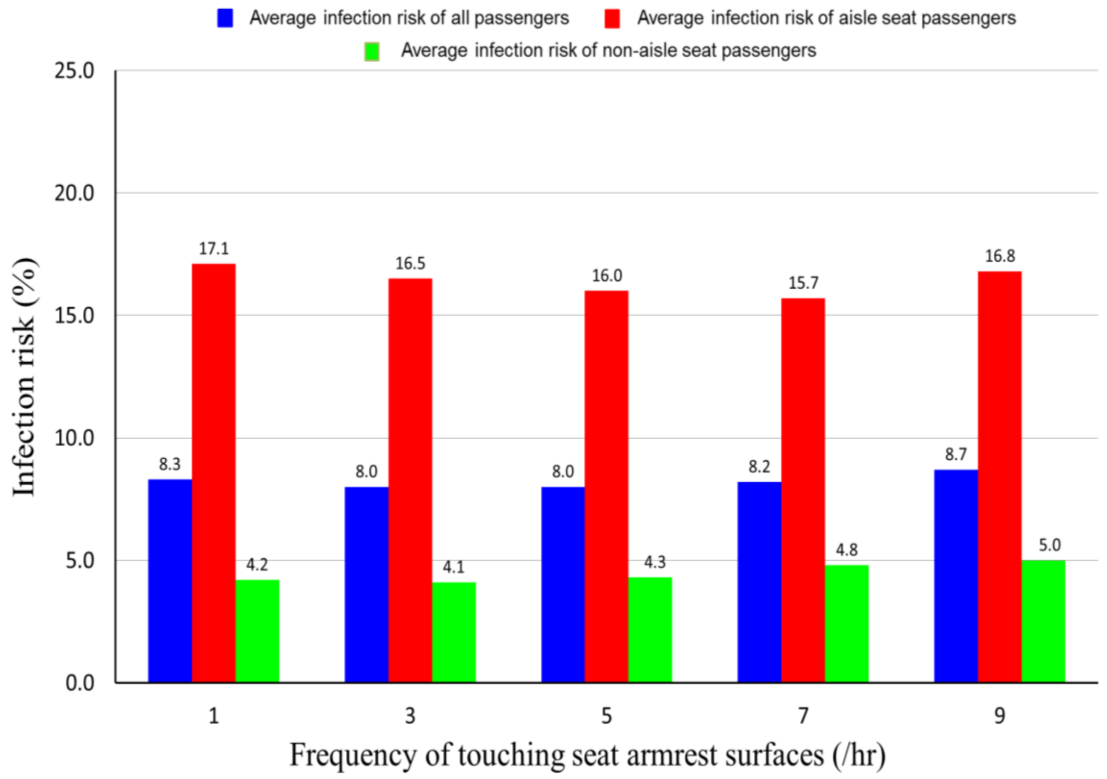

(d)

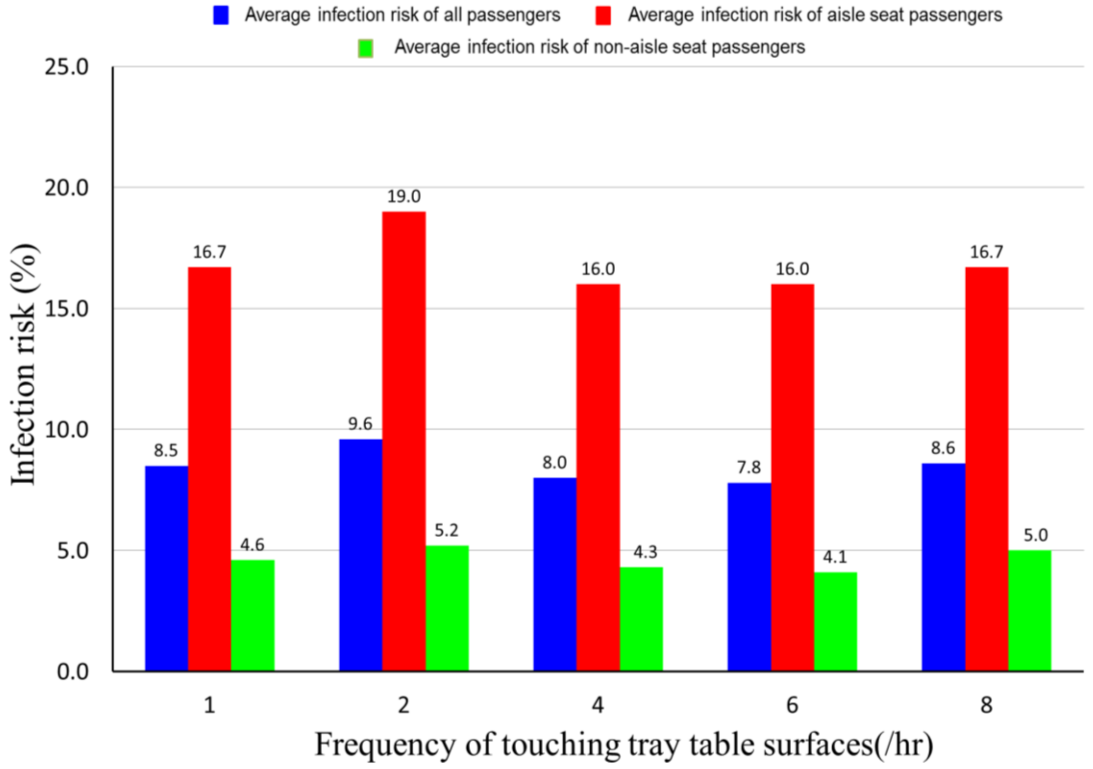

(e)

Figure S5 The average infection risk of all susceptible passengers via the fomite route: (a) as a function of toilet use frequency from 0.083 to 0.5 per hour, where the baseline value of this

parameter is 0.167 per hour; (b) as a function of the probability of touching the aisle seatback surfaces on the way to the toilet and back from 0.083 to 0.5, where the baseline value of this parameter is 0.167; (c) as a function of the frequency of touching the front seatback surfaces from 1 to 9 per hour, where the baseline value of this parameter is 3 per hour; (d) as a function of the frequency of touching armrest surfaces from 1 to 9 per hour, where the baseline value of this parameter is 5 per hour; (e) as a function of the frequency of touching tray table surfaces from 1 to 8 per hour, where the baseline value of this parameter is 4 per hour.

2.2 Supplementary properties of the surface contamination network

The infection source can be a toilet if an infector vomits in it, as in the GI 747 outbreak, or a contaminated seat surface where the infector sat, as in the GII 737 outbreak. If the toilet (W2) was contaminated first, the subsequent contamination process of aisle seatback surfaces is as shown in Figure S6. If the aisle seatback surface 14D was contaminated first, the surface contamination process is as shown in Figure S7. The aisle seatback surface contamination network is a directed network, in which the direction is determined by the surface touching sequence of each individual. Viruses on the surfaces can only be transferred in this direction. The touched surfaces are categorised by touch generation. The first surface(s) touched or contaminated by an infected person is the origin generation. The first generation is a group of surfaces touched by any individual whose hands are contaminated by touching the origin generation surface(s). In general, the nth generation is a group of surfaces touched by any individual whose hands are contaminated by touching the contaminated (n-1)th generation surface(s). The concept of generation is as important as infection risk. In general, the most recent generation surfaces have a lower virus concentration. In Figure S7, the aisle seatback surfaces in the first to the fifth are mainly located in the fourth and fifth generation of the surface contamination network, and we can qualitatively conclude that the aisle passengers sitting in the front of the cabin would have a lower exposure dose via the fomite route than others. The aisle passengers seated in the eleventh to twentieth rows would have a higher exposure dose via the fomite route than others because most of these rows are located in the first and second generation of the surface contamination network. This is consistent with the results from our computer simulation studies.

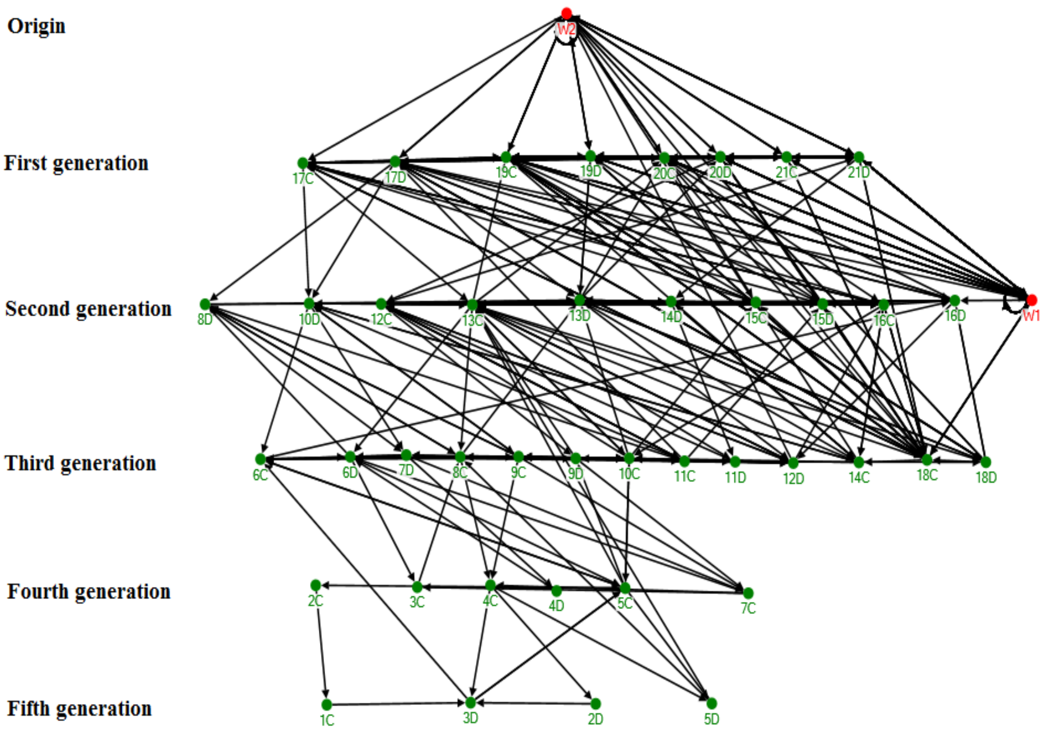

Figure S6. Surface contamination of aisle seatback surfaces after one toilet (W2) becomes contaminated.

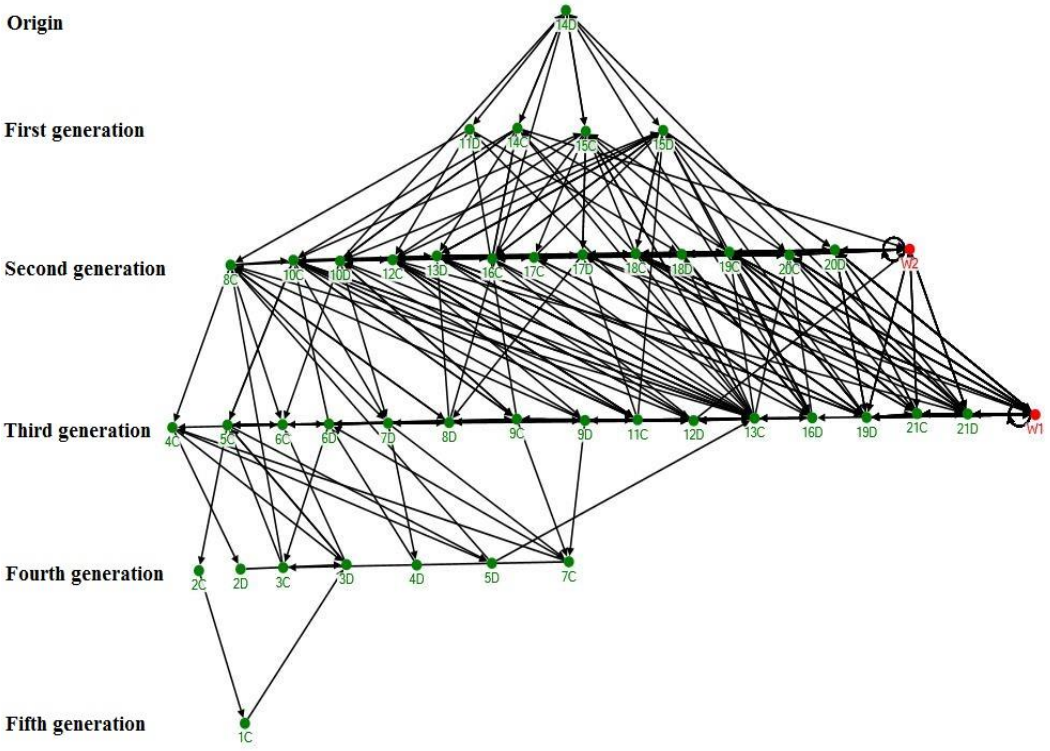

Figure S7. Surface contamination process after one seatback surface (14D) becomes contaminated.

2.3 The effect of different critical values of surface contamination

It is reasonable to assume that when the amount of GII genomes on surfaces is less than a critical value (e.g., more than 1 genome/cm^2), they cannot be transmitted to the contact hands. Therefore a critical number is suggested here for determining the minimum number of transmissible genomes on surfaces. The value of the critical number is most likely to be related to the surface material. For a rough surface such as fabric, this critical number may be high, whereas for a smooth surface such as glass, it can be much smaller. In this study, we adopt different values of the critical number for testing. Two values are tested (10 and 100 genomes/cm^2,) and the resulting number of contaminated surfaces is compared in Figures S8a,b and c,d. When the critical value is 10 genomes/cm^2, 65% of aisle seatback surfaces (26 of 40) will be contaminated, but only 40 of a total of 418 surfaces (9.5%) will be contaminated. This indicates that the aisle seatback surfaces are more contaminated, and so the aisle seat passengers are more likely to be infected. When the critical value is 100 genomes/cm^2, 9 aisle seatback surfaces and 11 total surfaces are contaminated, i.e., in addition to the 9 aisle seatback surfaces, 2 more surfaces are contaminated. It also indicates that aisle seat passengers are more likely to be infected via the fomite route. Whether the critical value is high (100 genomes/cm^2) or low (0 genome/cm^2), the R-square values are all larger than 0.97. Table S6 summarises the R-square and chi-square values of the four fits. When the number of degrees of freedom is large (Figures 3a,b and S8a,b), the chi-square value is large; the chi-square values in fits e and f are small because the number of degrees of freedom is small in these two fittings. The R-square and chi-square values of the fits indicate that the growth of the number of contaminated aisle seatback surfaces and of all surfaces both show a logistic trend.

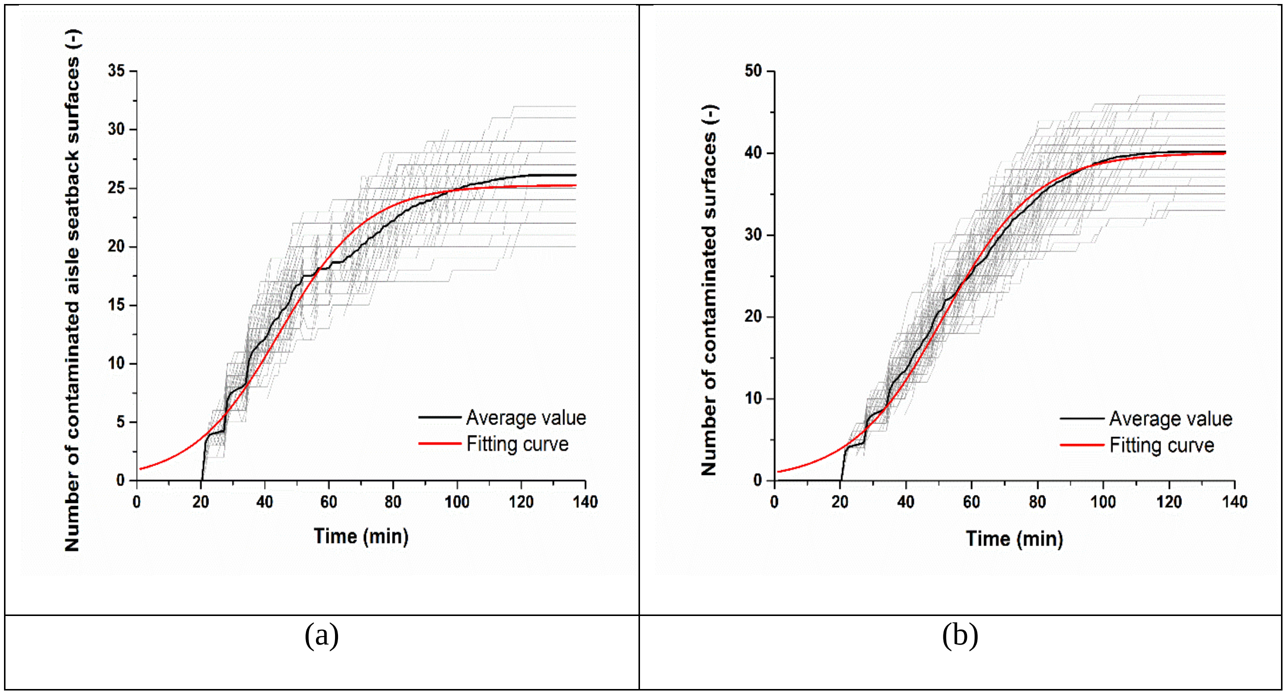

(a) (b)

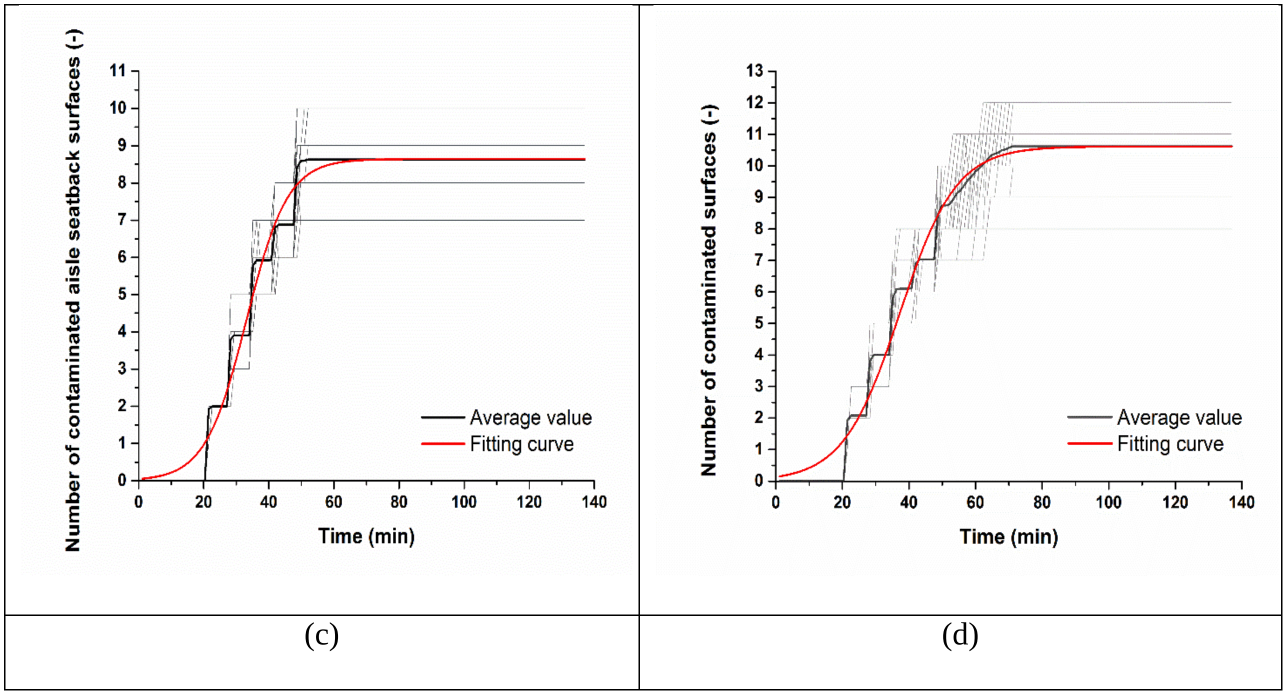

(c) (d)

Figure S8 Growth of the number of contaminated surfaces in the GII 737 outbreak, with different critical thresholds of transmissible genomes: (a), (b) 10 genomes/cm^2; (c), (d) 100 genomes/cm^2. Each graph shows all 100 simulation results (grey), the average of these 100 simulation results (black) and the fitting curve of the average value using the logistic function (red). The results for of 0 genomes/cm^{2}are shown in Figure 4 in the main text.

Table S6 R-square and chi-square in the logistic regression.

Fitting equation: $y = AB/(B + (A - B)e^{-cx})$		
	R^2	χ^2
Figure 3a	0.971	5.52
Figure 3b	0.994	70.83
Figure 3c	0.997	3.00
Figure 3d	0.998	131.72
Figure S9a	0.979	1.86
Figure S9b	0.993	1.51
Figure S9c	0.990	0.11

Figure S9d	0.991	0.15

Limitations of methodologies

This study has at least four limitations. First, although the airborne route plays a minor role, our simplifications in modeling the airborne route prediction need to be noted. The airborne droplet nuclei distribution in Economy class needs to be improved based on the understanding of the cabin airflow pattern. The focus of this study is on the surface contamination network related to the fomite route.Second, in the fomite route model, virus transfer by touch is always assumed to be from a high concentration surface to a low concentration surface. In practice, the transfer direction is most likely to be affected by the surface roughness and humidity. This may be addressed in future by developing an improved model of particle transfer between surfaces. Thirdly, due to a lack of data on the surface touching behaviour of passengers and crew members, the passenger behaviour patterns are assumed to be uniform. For example, all susceptible individuals are assumed to have the same toilet usage frequency. Further studies may be carried out in the aircraft cabin simulators or in real flights to acquire more information about human touching behaviours. Finally, also due to the lack of data, the initial norovirus concentration on all environmental surfaces was assumed to be 0 in both the computer simulations and the bench-top experiments, and the inactivation rate of norovirus in air was represented by that of the influenza virus.

REFERENCES

1. Kirking, H. L. *et al*. Likely transmission of norovirus on an airplane, October 2008. *Clin. Infect. Dis.* **50**, 1216-1221 (2010).
2. Holmes, J. D. & Simmons, G. C. Gastrointestinal illness associated with a long-haul flight. *Epidemiol. Infect.* **137**, 441-447 (2009).
3. Bean, B. *et al.* Survival of influenza viruses on environmental surfaces. *J. Infect. Dis.* **146**, 47-51 (1982).
4. Sattar, S. A. *et al.* Hygienic hand antiseptics: should they not have activity and label claims against viruses? *Am. J. Infect. Control* **30**, 355-372 (2002).
5. King, M. F. *et al.* Modeling environmental contamination in hospital single-and four-bed rooms. *Indoor Air*, **25**: 694-707 (2015).
6. Liu, P. B. *et al*. Persistence of human noroviruses on food preparation surfaces and human hands. *Food Environ. Virol.* **1**, 141-147 (2009).
7. Cannon, J. L. *et al*. Surrogates for the study of norovirus stability and inactivation in the environment: A comparison of murine *Norovirus* and feline *Calicivirus*. *J. Food Prot.* **69**, 2761-2765 (2006).
8. Sattar, S. A. *et al*. Transfer of bacteria from fabrics to hands and other fabrics: Development and application of a quantitative method using *Staphylococcus aureus* as a model. *J. Appl. Microbiol.* **90**, 962-970 (2001).
9. Mackintosh, C. A & Hoffman, P. N. An extended model for transfer of micro-organisms via the hands: differences between organisms and the effect of alcohol disinfection. *J Hyg (Lond).* **92**, 345-355 (1984).
10. Lopez, G. U, *et al.* Transfer efficiency of bacteria and viruses from porous and nonporous fomites to fingers under different relative humidity conditions. *Appl. Environ. Microbiol.* **79**, 5728-5734 (2013).

11. Rusin, P., Maxwell, S. & Gerba, C. Comparative surface-to-hand and fingertip-to-mouth transfer efficiency of gram-positive bacteria, gram-negative bacteria, and phage. *J. Appl. Microbiol.* **93**, 585-592 (2002).
12. Nicas, M. & Sun, G. An integrated model of infection risk in a health-care environment. *Risk Anal.* **26**, 1085-1096 (2006).
13. Hendley, J. O., Wenzel, R. P. & Gwaltney, J. M. Transmission of rhinovirus colds by self-inoculation. *N. Engl. J. Med*. **288**, 1361-1364 (1973).
14. Chan, M. C. *et al*. Fecal viral load and norovirus-associated gastroenteritis. *Emerg. Infect. Dis.* **12**, 1278-1280 (2006).
15. Glass, R. I., Parashar, U. D. & Estes, M. K. *Norovirus* gastroenteritis. *N. Engl. J. Med*. **361**, 1776-1785 (2009).
16. Nazaroff, W. W. *Norovirus*, gastroenteritis, and indoor environmental quality. *Indoor Air* **21**, 353-356 (2011).
17. Bonifait, L. *et al*. Detection and quantification of airborne norovirus during outbreaks in healthcare facilities. *Clin. Infect. Dis.* **61**, 299-304 (2015).
18. Marks, P. J. *et al*. Evidence for airborne transmission of Norwalk-like virus (NLV) in a hotel restaurant. *Epidemiol. Infect.* **124**, 481-487 (2000).
19. Cheesbrough, J. H. *et al*. Widespread environmental contamination with Norwalk-like viruses (NLV) detected in a prolonged hotel outbreak of gastroenteritis. *Epidemiol. Infect.* **125**, 93-98 (2000).
20. Wu, H. M. *et al*. A norovirus outbreak at a long-term-care facility: The role of environmental surface contamination. *Infect. Control Hosp. Epidemiol.* **26**, 802-810 (2005).
21. Nicas, M. & Jones, R. M. Relative contributions of four exposure pathways to influenza infection risk. *Risk Anal.* **29**, 1292-1303 (2009).
22. Barker, J., Vipond, I. B. & Bloomfield, S. F. Effects of cleaning and disinfection in reducing the spread of *Norovirus* contamination via environmental surfaces. *J. Hosp. Infect.* **58**, 42-49 (2004).
23. Chen, S. C., Chang, C. F. & Liao, C. M. Predictive models of control strategies involved in containing indoor airborne infections. *Indoor Air* **16**, 469-481 (2006).
24. International Commission Radiological Protection. Human respiratory tract model for radiological protection. *Ann. ICRP* **24**, 1-482 (1994).
25. Nielsen, P. V. *et al*. Contaminant flow in the microenvironment between people under different ventilation conditions. *ASHRAE Trans*. **114**, 632-640 (2008).
26. Cox, C. S. Aerosol survival of *Pasteurella tularensis* disseminated from the wet and dry states. *Appl. Microbiol.* **21**, 482-486 (1971).
27. Ehresmann, D. W. & Hatch, M. T. Effect of relative humidity on the survival of airborne unicellular algae. *Appl. Microbiol.* **29**, 352-357 (1975).
28. Riley, R. L. & O'Grady, F. Airborne infection: transmission and control. (The Macmillan Company, 1961).
29. Xie, X. *et al*. Bacterial survival in evaporating deposited droplets on a Teflon-coated surface. *Appl. Microbiol. Biotechnol.* **73**, 703-712 (2006).
30. Xie, X. *et al*. How far droplets can move in indoor environments: revisiting the Wells evaporation-falling curve. *Indoor Air* **17**, 211-225 (2007).
31. Jones, R. M. *et al*. Characterizing the risk of infection from *Mycobacterium tuberculosis* in commercial passenger aircraft using quantitative microbial risk assessment. *Risk Anal.* **29**, 355-365 (2009).

32. Harper, G. J. Airborne micro-organisms: survival tests with four viruses. *J. Hyg.* (Lond) **59**: 479-486 (1961).
33. Thatcher, T. L. *et al*. Effects of room furnishings and air speed on particle deposition rates indoors. *Atmos. Environ.* **36**, 1811-1819 (2002).
34. Atkinson, M. P. & Wein, L. M. Quantifying the routes of transmission for pandemic influenza. *Bull. Math. Biol.* **70**, 820-867 (2008).
35. Thebault, A. *et al*. Infectivity of GI and GII noroviruses established from oyster related outbreaks. *Epidemics* **5**, 98-110 (2013).
